# Adsorptive Cathodic Stripping Analysis of Xylazine Within Fouling-Resistant and Nanomaterial-Enhanced Modified Electrode Sensors

**DOI:** 10.3390/s25175312

**Published:** 2025-08-26

**Authors:** Michael C. Leopold, Charles W. Sheppard, Joyce E. Stern, Arielle Vinnikov, Ann H. Wemple, Ben H. Edelman

**Affiliations:** Department of Chemistry, Gottwald Center for the Sciences, University of Richmond, Richmond, VA 23173, USA; charlie.sheppard@richmond.edu (C.W.S.); joyce.stern@richmond.edu (J.E.S.); arielle.vinnikov@richmond.edu (A.V.); holly.wemple@richmond.edu (A.H.W.); ben.edelman@richmond.edu (B.H.E.)

**Keywords:** fentanyl, xylazine, adsorptive, cathodic, stripping analysis, modified electrode

## Abstract

Xylazine (XYL), an FDA-approved veterinary tranquilizer, is being abused both as an opioid adulterant in a street-drug known as “Tranq-dope” and as a date rape drug. Given its now nearly ubiquitous use with fentanyl and fentanyl derivatives across the globe, XYL has become a primary target for researchers seeking to develop portable and cost-effective sensors for its detection. Electrochemical sensors based on the oxidation of XYL, while useful, have limitations due to certain interferents and inherent electrode fouling that render the approach less reliable, especially in certain sample matrices. In this work, modified electrode platforms incorporating layers of multi-walled carbon nanotubes for sensitivity along with semi-permeable polyurethane (PU) layers and host–guest chemistry using β-cyclodextrin for selectivity are deployed for XYL detection using complementary adsorptive cathodic stripping analysis. The modified electrode sensors are optimized to minimize high potentials and maintain fouling resistant capabilities and investigated to better understand the function of the PU layer. The use of adsorptive cathodic stripping differential pulse voltammetry indirectly indicates the presence and concentration of XYL within complex sample media (beverages and synthetic urine). When used in this manner, the modified electrodes exhibited an overall average sensitivity of ~35 (±9) nA/μM toward XYL with a limit of quantification of <10 ppm, while also offering adaptability for the analysis of XYL in different types of samples. By expanding the capability of these XYL sensors, this study represents another facet of tool development for use by medical professionals, first-responders, forensic investigators, and drug-users to limit exposure and help stem the dangerous and illegal use of XYL.

## 1. Introduction and Background

As a highly potent analgesic opioid medication, fentanyl was created for use as an anesthetic drug [1,2]. Fentanyl and its numerous synthetic analogs, with efficacy and toxicity significantly higher than morphine and heroin, have also spread across the globe in the illicit drug market and contributed to the now widely recognized public health threat. The incredibly high potency of this drug has led to massive numbers of overdose deaths in recent years [1,2,3,4,5,6]. For example, a 2021 U.S.-based study [3] showed nearly 70% of the drug overdose deaths (107,521) involved fentanyl or a derivative thereof. That study echoes the larger trends over the last decade, which show an exponential increase in overdose victims, with a vastly disproportionate number being linked to fentanyl-class synthetic opioids [3]. Given the clear danger and societal cost of these illicitly marketed and highly abused opioids, fast and accurate detection of such compounds becomes critical for protecting people that have risk of exposure: drug-users or victims, first-responder medical personnel, border agents seeking to prevent the substance from crossing borders, and forensic investigators [2,7].

Scientific sensor research targeting synthetic opioids like fentanyl has been the subject of intense study over the last several years [7,8,9,10,11], including a particular focus on the development of electrochemical sensors [12,13,14,15,16], as they offer more cost-effectiveness, ease-of-operation, design adaptability, and portability [16]. Studies focused on electrochemical sensors for fentanyl and/or one or more of its derivatives are prevalent in the literature [17,18,19,20]. Included in this body of work is the strategy of incorporating nanomaterials (NMs) into sensing schemes to improve signal-to-noise ratios and subsequently achieve lower limits of detection quantification (LOQ) and detection (LOD) [7,21,22]. Major challenges still exist for researchers aiming to develop effective “fentanyl” sensors, with a major problem being the sheer number of synthetic iterations available and in circulation. A 2024 study reported finding more than 1400 fentanyl derivatives in the scientific literature [3], with the vast majority of derivatives having only minor structural differences but maintaining extremely high potency and lethality. While minor, these structural differences make it difficult to develop sensors with selectivity that accommodates the variability of the target molecules [23,24].

An extraordinary exasperation of the opioid crisis has been the rapid and voluminous emergence of xylazine (XYL)-adulterated fentanyl and its derivatives [25,26,27,28,29,30]. XYL or N-(2,6-dimethylphenyl)-5,6-dihydro-4H-1,3-thiazin-2-amine is FDA-approved for veterinary use as a non-opioid sedative and muscle relaxant (i.e., tranquilizer), marketed and commercially available under names such as Rompun^®^, Sedazine^®^, Chanazine^®^, Proxylaz^®^, and Anased^®^ [2,27,31]. Drug traffickers increase profits by adulterating or “cutting” fentanyl-based opioids with XYL, creating a mixture that delivers similar euphoric effects for users over longer periods while enabling broader distribution of actual fentanyl supplies [28]. The combination of fentanyl opioids with XYL has become a widespread and highly lethal street drug known as “Tranq dope” [2]. The U.S. Drug Enforcement Agency recently reported that “Tranq”, formulated as powder, injectable liquid, and pills, is the dominant abused drug in 48 out of 50 states [27,32,33]. Tranq dope has proven catastrophic in the opioid crisis, rapidly spreading throughout communities and dramatically escalating overdose deaths. In only one year (2020–2021), postmortem XYL detection in overdose victims drastically rose in all geographic regions of the U.S., including increases of 103% in the Northeast, 516% in the Midwest, 750% in the western states, and a staggering 1127% across southern states [32,33,34]. The damage caused by adulterated fentanyl also includes major medical conditions for users that improperly inject the substance, which causes severe skin ulcers and flesh necrosis, often leading to disabled limbs and/or requiring amputations [35,36]. The prevalence of these adverse effects has even earned the drug cocktail the additional street name of “Zombie” [2]. XYL sedative effects are unresponsive to naloxone (Narcan^®^), a counter-treatment of opioid poisoning and there is no human-approved antidote to reverse XYL intoxication. As such, first-responders can be easily enticed to over-deliver naloxone on unresponsive victims, often unaware of the adulterant [27,28].

The toxic/lethal levels of XYL exposure are not clear, as the drug has never been approved for human use and limited data are available. Even in its approved use as a veterinarian tranquilizer, the situation is complicated by the fact that the recommended dosages vary greatly by species. The inherent bioavailability once introduced into the animals varies greatly by species and by the methodology of drug delivery (i.e., intravenously (IV) vs. intramuscular (IM) or subcutaneous (SC) injections). For example, the bioavailability of XYL after IM injection in horses, sheep, and dogs are 40–48%, 17–73%, and 52–90%, respectively. A consequence of these varying ranges is that the onset of the drug’s action, its half-life, as well as recovery times also have a wide range. The onset of XYL effects can be 1–2 min in horses while being significantly longer in dogs and cats (~15–30 min) [37]. Recommended dosages, like most drugs, are based on the animal’s weight. In the case of a dog, one of the more relatable animals it is used on, the recommended safe dosage is 1.1 mg/kg, introduced via IV or 1.1 to 2.2 mg/kg if injected (IM/SC) [37]. Importantly, it should be recognized that XYL is designed to be delivered through IV introduction or IM/SC injection. While the injection toxicity may be somewhat relevant to XYL as an adulterant in fentanyl, it has unknown bearing on its bioavailability if ingested, as in the case of its use as a date rape drug (spiked drink). All these variables make it difficult to definitively determine toxic and/or lethal levels of human exposure. A 2023 review showed a wide XYL range (40 to 2400 mg) was reported as toxic and/or fatal for humans. Studies of XYL in blood showed a range from 0.03 to 4.6 mg/L in non-fatal cases, while postmortem analysis of blood yields a concentration range from trace levels to 16 mg/L that clearly overlaps [38]. Given that there is no way of knowing what level of XYL would be used in a nefarious or drug abuse situation, the prudent measure for detection is to consider that nearly any appreciable amount of XYL is dangerous.

The need for the fast, on-site, and accurate detection of Tranq or the presence of low levels of XYL in beverages is clearly a worthy scientific goal [9,34]. The nearly ubiquitous presence of XYL with fentanyl drugs means that XYL detection by a sensor likely indirectly indicates the presence of fentanyl or a fentanyl derivative, one of the few advantageous aspects of the fentanyl adulteration escalation. Rapid and versatile detection of XYL in different media such as beverages and urine could also be the premise of a tool for combating the illegal use of XYL as a “date rape” drug [39,40,41,42,43]. While significantly less prominent compared to fentanyl, reports of studying XYL electrochemistry and/or XYL sensor development [31,44], including the incorporation of NMs into sensing schemes [42,43,45,46,47], are emerging in the literature. This prior work is foundational, as it identifies important challenges in the area, including that no clear mechanism for XYL electrochemistry, particularly beyond the initial oxidation (below) of the compound, is agreed upon [31,41,42,43,45,48]. The limited number of papers examining electrochemical detection of XYL focus exclusively on XYL oxidation, with a general oxidation mechanism (below) [42,43,45]: 
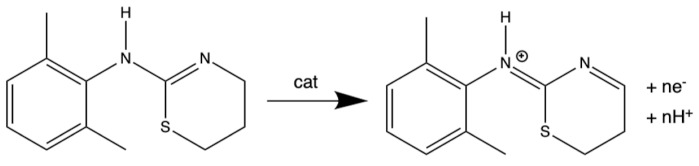


However, other redox mechanisms have been proposed as well [31,41,48]. Work by Mendes et al. not only establishes XYL electroactivity at different electrode interfaces but projects mechanistic details beyond initial oxidation to include adsorbed oxidation products [48]. Deciphering these electrochemical mechanisms is clearly complex, and while *not* the focus of our study, it was intriguing to consider the redox chemistry of that adsorbed oxidation product (see below).

Within the limited number of XYL electrochemical studies published, there seems to be consensus that XYL oxidation results in surface fouling of bare electrodes by that oxidation product [31,41,42,43,45,46,47,48]. The fouling commonly manifests as a blocked electrode [48], resulting in non-linear calibration curves [31,45] or curves with two distinct linear regions attributed a fouled electrode interface [42,43], as well as systematic shifts in peak potentials during XYL exposure [41,42,43]. These studies offer strategies to mitigate electrode fouling toward effective XYL analysis, including, for example, electrode repolishing after XYL exposures [31,48], specific fouling *resistant* electrode modifications [46,47], and/or application of pulsed (vs. sweep) electrochemical methods [42,43,45,46,47]. While all have attributes, some of these approaches require extensive pretreatments [41,42] and/or are impractical for portable measurements [31]. In critiquing our own studies of oxidative XYL sensors, amperometric schemes suffer from single-order (channel) dimensionality that can mask interferents in complex matrices, while sensitivity suffers from extended application of high oxidative potentials that can exasperate fouling [46]. Alternatively, while voltametric examination of XYL oxidation improves interferent detection and lessens the opportunity of fouling, the invocation of high positive potentials invites additional interferent responses in certain complex matrices [31,42,43,47,48]. Limitations of those approaches included that the XYL oxidation signal precluded its detection in certain sample media, including certain beverages as well as simulated urine. An alternative approach, applying minimal high oxidative potentials and utilizing an XYL signal at a different potential, while still limiting electrode fouling, may accommodate interferences and expand the sample matrices that can be analyzed.

Adsorptive stripping voltammetry is a well-established technique for electrochemical analysis [49,50] and has been applied for XYL sensing as well. The Merli lab recognized the value of XYL preconcentration in its electrochemical analysis in a laboratory-based, solid-phase extraction procedure [31], while the most advanced electrochemical study of this nature by Limbut et al. (2022) focused on anodic adsorptive stripping of XYL oxidation [42]. In our current study herein, XYL electrochemical analysis is reimagined using established modified electrodes that merge fouling resistance with adsorptive *cathodic* stripping voltammetry, allowing for minimal application of high oxidative potentials and an alternative methodology to avoid certain interferents that plagued oxidative XYL sensors. This study establishes the quantitative effectiveness of the sensor and postulates a redox mechanism to explain its functionality and observed behavior.

## 2. Experimental Details

### 2.1. Materials and Instrumentation

Electrochemical instrumentation from CH Instruments (Bee Cave, TX, USA) were utilized for this study. Specifically, 8-channel potentiostats (Models 1000B or 1030C), along with glassy carbon electrodes (GCEs), Ag/AgCl (satrd. KCl) reference electrodes from CH Instruments, and counter electrodes of coiled platinum wire (Millipore-Sigma, St. Louis, MO, USA) were employed. All chemical materials were purchased from commercial vendors in high purity and used as received in solutions with ultra-purified water (18.2 MΩ∙cm). Electrode modification materials included polyurethanes of hydrothane (HPU, AL25-80A) and Tecoflex (TPU, SC-80A), received from AdvanSource Biomaterials (Wilmington, MA, USA) and Lubrizol (Cleveland, OH, USA), respectively. Multi-walled carboxylic-acid-functionalized carbon nanotubes (COOH-MWCNT) were obtained from Nano Lab Inc. (Waltham, MA, USA), in conjunction with β-cyclodextrin (β-CD) purchased from Ambeed, Inc. (Arlington Heights, IL, USA). Chem-Impex International (Wood Dale, IL, USA), through VWR International LLC, was the source of xylazine (XYL) for this study. Simulated urine solutions (Sigmatrix Urine Diluent) were purchased from Oakwood Chemical (Estill, SC, USA), while bourbon whiskey (Wild Turkey^TM^) was obtained from the nearby ABC store. Hard seltzer (White Claw^TM^, peach) was purchased at the local Publix grocery store (Richmond, VA, USA). Transmission and scanning electron microscopy imaging was performed on a JEOL 1010 Microscope with an Advanced Microscopy Techniques XR-100 Digital CCD and JEOL 6360 Low Vacuum Microscope (Peabody, MA, USA).

### 2.2. Solutions and Materials Preparation

Prior to fabricating the modified electrodes (see below), several solutions were created, including 150 mM potassium phosphate buffer or PBS (pH = 7.0), a polyurethane (TPU), and a COOH-MWCNT and β-CD mixture. A neutral pH buffer was selected, given the optimal XYL oxidation behavior established in the Mendes study [48]. The PU material, typically either in ratios of 0:100 or 75:25 HPU–TPU, was prepared by adding the corresponding mass (100 mg TPU, or 75 mg and 25 mg of HPU and TPU, respectively) to a 1:1 ethanol–THF solvent mixture (5 mL) that was then stirred overnight and sonicated, if necessary, for complete dissolution [46,47,51]. Note: It is critical that the PU component is completely dissolved without using heat to avoid high variance in results. The COOH-MWCNT and β-CD mixture contained 6 mg of each material in 200 proof ethanol (3 mL), which was subsequently sonicated for 30 min. Importantly, these solutions were freshly prepared for each experiment on a weekly basis and refrigerated (7 °C) until use. Sonication and the wt % ratio of COOH-MWCNT and β-CD were optimized in prior studies [52,53]. TEM imaging of dispersed COOH-MWCNT with β-CD and SEM of GCE modified with the same material are provided in Supplementary Materials (Figure S1), all showing typical structure and morphology.

### 2.3. Sensor Fabrication

Sensor fabrication proceeded as described in previously reports utilizing similar modified electrode schemes [46,47]. Deposition of materials at different stages of the electrode modification were previously characterized using IR spectroscopy, chronocoulometry, and voltammetry, as well as various microscopy techniques (SEM, AFM, TEM), some of which were repeated for this study (Supplementary Materials, Figure S1) [52,53,54]. Briefly, GCEs were freshly polished with sequential application of Al_2_O_3_ slurries (1.0, 0.3, and 0.05 μm) on microcloths (Buehler, Lake Bluff, IL, USA) and were used immediately for all experiments. In the event a polished electrode was not used immediately, it was repolished (0.05 μm Al_2_O_3_) before its use. After excessive rinsing of polished electrodes, they were dried under a N_2_ stream and inspected for any surface anomalies (Note: damaged electrodes were repolished or discarded). A micropipette was used to deposit the COOH-MWCNT and β-CD mixture (7 μL) and allowed to dry under ambient conditions (10 min), followed by PU deposition (10 μL) and a second ambient drying period (10 min). Modified electrodes were then allowed to soak in PBS for 15 min before proceeding.

### 2.4. Sensor Operation

Buffer-equilibrated modified electrodes were transferred to the electrochemical cell as the working electrode in fresh PBS solution (25 mL) for testing. Differential pulse voltammetry (DPV) scans toward either positive or negative potentials were conducted on PBS solutions (background) or PBS solution with added XYL. DPV experiments were conducted with the following parameters: potential increment of 4 mV, amplitude of 0.07 V, pulse width of 0.05 s, sample width of 0.0167 s, and a pulse period of 0.5 s. Certain DPV experiments were conducted to maximum oxidative and reductive potentials of +1.5 V to −0.8 V, respectively. The most typical potential range was +1.1 V to −0.4 V, as established in the potential dependence section (below). Unless otherwise stated, experiments designed to establish properties of the modified electrodes were typically conducted with XYL injections followed by 10 s vigorous stirring (1000 rpm) for mixing and then a minimum of 3 min of rest time prior to applying the potential. For some experiments, DPV scans were started immediately to capture certain film behaviors. In general, DPV scans were run in quiescent solutions with diffusion as the only mass transport mode. During DPV stripping (DPSV) analysis, the “stripping mode” of the potentiostat was utilized, allowing for an initial potential (E_init_) and a holding time to be selected prior to the DPV cathodic sweep—synonymous with a deposition potential in more traditional stripping voltammetry (e.g., Hg drop electrodes). Peak currents and areas were corrected by manually removing the baseline charging current. Analysis of spiked beverages and synthetic urine were approached from a sampling standpoint, as in reports [46,47], in that calibration curves (CCs) were collected in PBS with sequential addition of XYL standard and cathodic DPV scans. After calibration, the electrodes were thoroughly rinsed with ultra-purified water before transfer to a mixture of PBS spiked with 0.5 mL of the media being tested (e.g., synthetic urine) for at least 3 min, in order to allow for XYL, both its oxidized and subsequently reduced species, to leech from the film prior to immersion and cathodic scanning in 127 μM XYL-spiked media, a response measured against the standard curve for percent recovery. Measurements of pH were conducted prior to and after full analysis to ensure that buffer capacity had not been exceeded.

## 3. Results and Discussion

### 3.1. Electrochemical Behavior of XYL at GCEs

**Figure 1:**
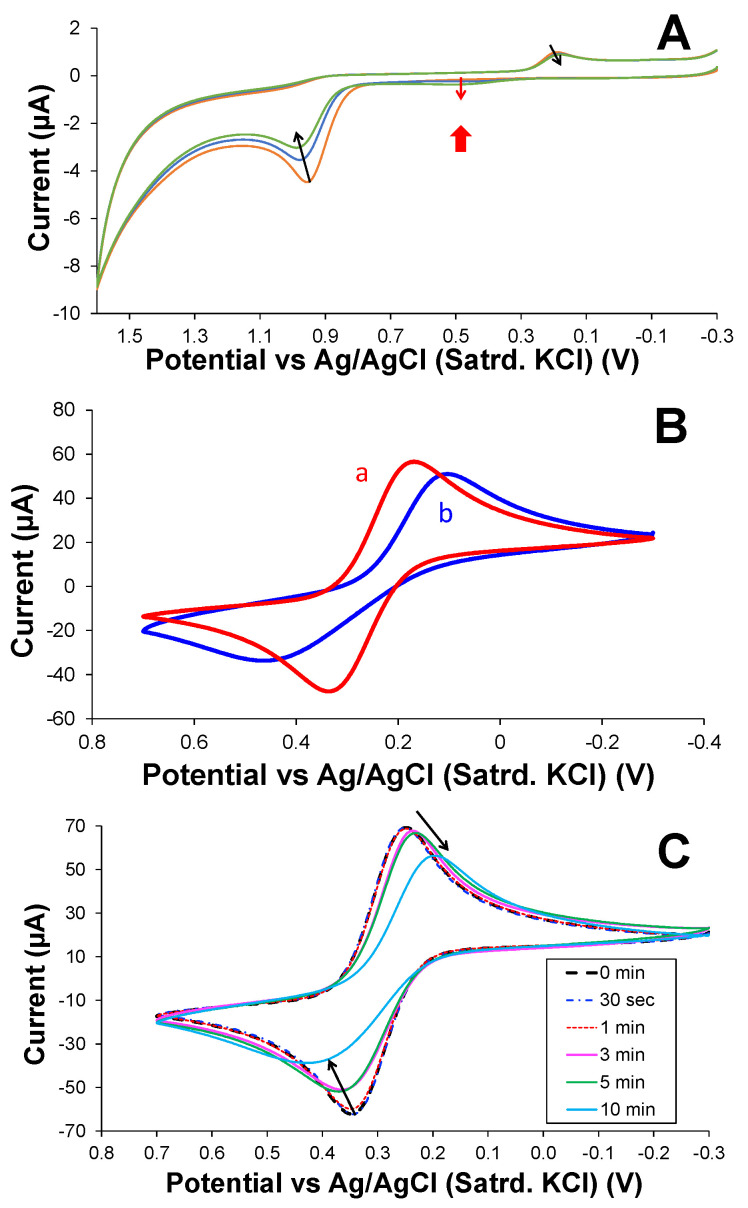
(**A**) Three successive cyclic voltammetry (CV) scans of an unmodified GCE immersed in 1 mM XYL (150 mM PBS at pH = 7); red arrow at ~0.5 V highlights an anodic process that appears only after the first scan and then slowly increases with each subsequent scan, an indication of immediate electrode fouling after XYL oxidation [46,47,48]; (**B**) CV of 5 mM potassium ferricyanide (K_3_Fe(CN)_6_) in 0.5 M KCl at a bare GCE both (a) before and (b) after extensive XYL oxidation exposure imposed by prior application of +1.1 V in 1 mM XYL solution; and (**C**) similar CV of K_3_Fe(CN)_6_ on a set of GCEs with different application times of the +1.1 V in 1 mM XYL solution showing the fouling is intrinsically linked to XYL oxidation. Notes: Scan rate of CV is 100 mV/s.

XYL redox behavior at an unmodified glassy carbon electrode (GCE) during successive scans of cyclic voltammetry (CV) is shown in Figure 1A. During repeated cycling, the commonly reported XYL electrochemical behavior can be observed: irreversible voltammetry featuring a prominent peak ~+0.95 V during XYL oxidation and subsequent reduction of that oxidation product at ~+0.2 V, with both peak currents diminishing with each subsequent cycle as the interface fouls and shifts the peak potential [46,47,48]. Notably, the reductive wave at +0.2 V is always smaller than the oxidation wave during each cycle. Also, a second anodic peak consistently emerges at (~+0.5 V) after the first cycle, suggesting material is being adsorbed to the electrode surface. This result is consistent with the literature reports and attributed to electrode fouling related to XYL oxidation. Prior scan rate analysis of these peaks show mixed diffusional/adsorbed behaviors that are unsurprising given the permeable nature of a fouled electrode system [47,48].

Examining the CV of potassium ferricyanide (K_3_Fe(CN)_6_) in the solution at GCEs that have been subjected to different XYL exposures was instructive. Figure 1B shows the CV of K_3_Fe(CN)_6_ before and after significant XYL exposure during amperometric current–time experiments, where five successive injections (50 μL) of 50 mM XYL standard every 200 s were made under a +1.1V constant applied potential (Supplementary Materials, Figure S1) [46]. After extensive XYL exposure, subsequent K_3_Fe(CN)_6_ CV shows the exposed electrode interfaces are significantly altered compared to the response at a bare GCE. Prior to XYL exposure, the K_3_Fe(CN)_6_ voltammetry exhibited an expected diffusional peak shape with fast electron transfer kinetics (Figure 1B, trace a). After the exposure to XYL oxidation, however, the response transforms to irreversible voltammetry, suggestive of a significant blocked electrode interface (Figure 1B, trace b), strong evidence of a significantly fouled electrode. Results from a different systematic study linking electrode fouling to the amount of XYL oxidation is captured in Figure 1C, where holding the electrode at +1.1 V in a XYL solution for different amounts of time prior to DPV sweeps leads to the same type of K_3_Fe(CN)_6_ voltammetry shifts. A similar effect was observed when K_3_Fe(CN)_6_ voltammetry was collected on electrodes that were immersed in XYL solution and simply scanned at different scan rates, with slower scan rates allowing for more significant XYL oxidation and producing more blocked interfaces (Supplementary Materials, Figures S3 and S4).

**Figure 2:**
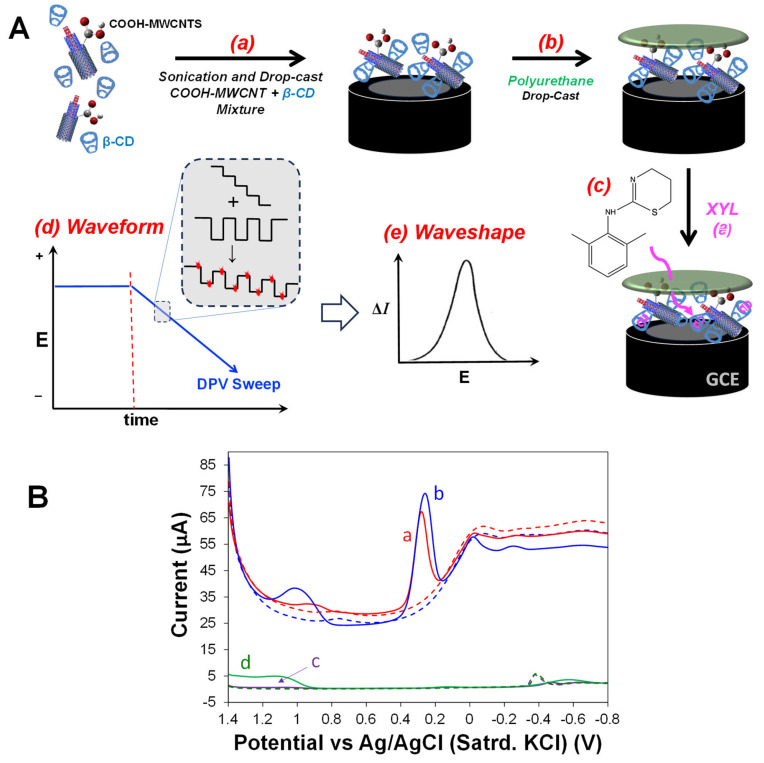
(**A**) Schematic illustration of GCE modification including sonication and drop-cast of (a) COOH-MWCNT (sensitivity) with β-CD (selectivity); (b) drop-cast of PU capping layer (selectivity); (c) immersion in sample with XYL followed by (d) application of stripping DPV waveform (sensitivity); and (**e**) the resulting voltammetry. (**B**) Cathodic DPVs at of fully modified GCEs (75:25 PU capping layer over COOH-MWCNTs with β-CD immersed in (a) 1 mM XYL and (b) 5 mM XYL (150 mM PBS; pH = 7) and corresponding background scans (dashed traces) in PBS (0 mM XYL) along with analogous scans (c,d) in the same solutions with GCE modified with only the PU capping layer.

For research aiming to develop XYL sensors, the complexity of this electrochemistry has prompted a focus on the initial XYL oxidation peak, sometimes coupled with strategies to compensate for the fouling (e.g., GCE repolishing after each XYL exposure) [31,48]. One mitigation strategy was the use of layer-by-layer modification of the GCE interface to improve fouling resistance and provide enhanced sensing functionality [46,47]. That interface, however, still utilizes XYL oxidation as the main detection signal, making fouling a concern for long-term use and precluding its use in situations where there are interferents with similar oxidation potentials. In the current study, the modified electrode scheme developed in those previous studies (Figure 2A, scheme) [46,47], GCEs modified with carboxylic acid multi-walled CNTs (COOH-MWCNTs) for sensitivity enhancement, and β-cyclodextrins (β-CDs) and a polyurethane (PU) layer, both for improved selectivity, are again utilized [46,47,55,56]. NMR studies [55,56] established host–guest interactions between XYL and the β-CD utilized in the scheme that proved critical to enhancements in sensitivity and selectivity [46,47]. Selectivity of these modified electrodes showed explicit discrimination against a number of notable potential individual interferents found in beverages (e.g., caffeine, sugars, artificial sweeteners, citric acid) [46,47], as well as interferent compounds commonly found adulterated by XYL (e.g., cocaine and fentanyl) [47]. As such, those aspects of this electrode scheme are not revisited herein and are instead accepted. The current study focuses on how the modified interfaces can be expanded for additional and complementary XYL analyses, particularly in sample media with multiple interferents that prohibit an oxidative approach. This study represents the first of its kind exploring cathodic electrochemistry related to XYL.

Figure 2B shows differential pulse voltammetry (DPV) of GCEs, modified as shown (Figure 2A) and immersed in solutions of 5 mM, 1 mM, and 0 mM XYL before being scanned in a cathodic direction. Because the scans are initiated at potential (+1.4 V), XYL is readily oxidized at the start of the scan and that oxidation product is then subsequently reduced or cathodically stripped from the film to produce a large peak at +0.3 V during the latter parts of the DPV sweep. Even though the reductive DPV peak remains smaller than its apparent oxidative counterpart, the signal is sensitive to different XYL concentrations. Additionally, it is evident that the COOH-MWCNTs with β-CD enhances the signal, while the PU itself seems to significantly passivate the electrode interface. DPV is well known to be more sensitive than sweeping electrochemical techniques (e.g., CV), and the fully modified electrode clearly exhibits an increase in both the cathodic signal as well as the background current, a phenomenon that needed further investigation before exploiting the system for XYL detection. For reference, CV results of bare and fully modified GCEs (Supplementary Materials, Figures S5 and S6) re-emphasize these observations.

### 3.2. Electrochemical Effects of the PU Capping Layer

Use of hydrophobic and hydrophilic PU materials as an outer, semi-permeable membrane for sensing schemes is a well-established strategy, often resulting in improved sensor selectivity but sometimes at the expense of sensitivity [57]. The functions of the PU layers in this study are no different. Herein, PU from both ends of the hydrophobicity spectrum are explored for XYL sensing, with a focus on either 75:25 or 0:100 of HPU (hydrophilic)-to-TPU (hydrophobic) ratios. The selection of these specific PU blends was based on prior reports that optimized similarly modified electrodes for the oxidation of XYL. While the HPU–TPU ratio of 75:25 was found to be optimal for XYL permeation, it was also susceptible to interferents and limited in its application to certain matrices [46,47]. The demonstration of our sensor functionality in this work using both the 75:25 PU blend as well as more hydrophobic film ratio (100:0) is considered a feature of the sensing scheme, offering a hydrophobicity-based parameter that can be specifically adapted for different media and the interferents present to improve overall selectivity. Additionally, the PU layer sustains the sensing scheme, in that it adds robustness to the electrodes, preventing loss of MWCNTs from the film [46,47,51]. That said, selection of the PU does have an important and notable effect on background signal during XYL measurements that may suggest information about the movement of species into and within the layered modification.

**Figure 3:**
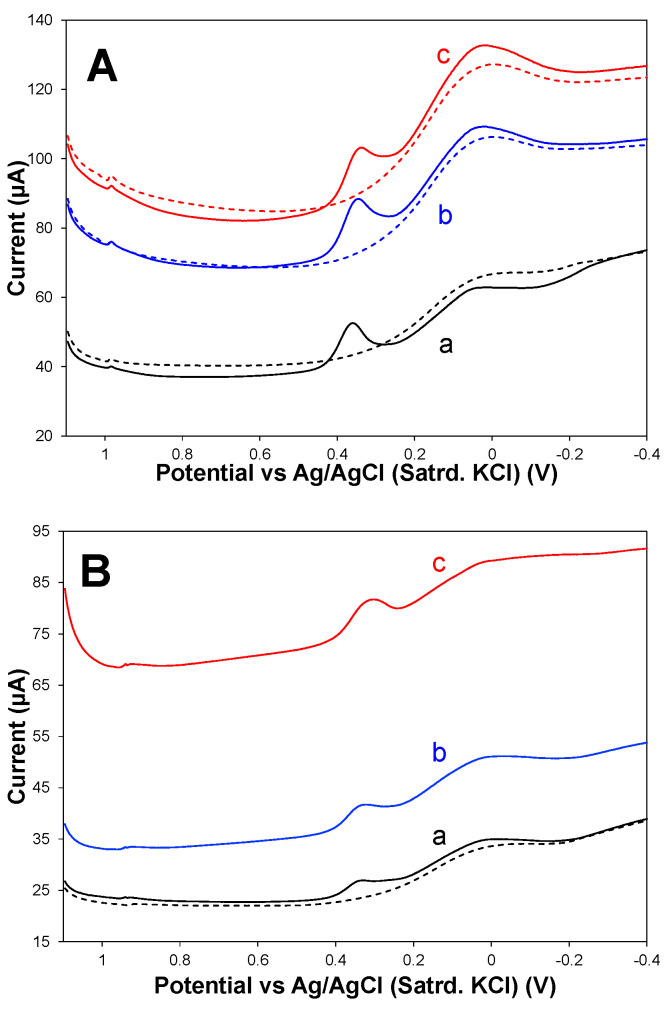
Cathodic DPV scans in 300 μM XYL (150 mM PBS; pH 7) and corresponding backgrounds for (**A**) modified electrodes capped with (a) 0:100, (b) 50:50, and (c) 75:25 HPU–TPU blends used as the capping layers, and (**B**) modified electrodes with 75:25 HPU–TPU capping layers with different deposition volumes of the COOH-MWCNTs and β-CD mixture: (a) 5, (b) 7, and (c) 15 μL (Note: dashed trace represents background in PBS with 0 mM XYL).

Layer-by-layer electrode schemes are well known to invoke both changes to the signal (Faradaic current) and background (non-Faradaic or charging current), the latter often affected by changes in the electric double layer at the electrode interface and/or redefining the system’s diffusional layer [58]. Similarly, the incorporation of nanoparticles (e.g., CNTs) are also known to affect both signal and noise/background currents as well [46,47,51]. The behavior of the electric double layer at an electrode interface, the double layer capacitance (C_dl_) of the film, mimics a parallel-plate capacitor model obeying the following relationship:

(1) $$C_{\mathrm{dl}}\;\approx \;\frac{\varepsilon \cdot \varepsilon_{{}^{\circ}}}{d},$$

where ε_o_ is the permittivity of the free space constant, while ε and d represent the dielectric constant (polarizability) of the material and distance separating the planes of charge between the polarized electrode and counter-charges in the solution at that interface, respectively [58,59,60]. The interplay of these variables helps explain notable background current observations in this study. For example, as illustrated in Figure 3A, the background current during DPV with and without XYL present shows it to be a function of the amount of the HPU component used in the capping layer. As such, systems utilizing a blend of 75:25 HPU–TPU will have higher charging currents than those using 0:100 HPU–TPU capping layers, with the XYL cathodic signal from 300 μM XYL DPV sweeps rising above that background. Similarly, when a film capped with either 75:25 or 0:100 HPU–TPU is immersed in XYL solution for an extended time, the XYL permeation and its infusion throughout the film results in a slight decrease in background current over time (Supplementary Materials (Figure S7)). An additional complicating factor, however, is that both background and XYL signals increase as a function of higher amounts of COOH-MWCNTs present beneath the PU capping layers (Figure 3B and Supplementary Materials, Figure S8). The incorporation of NMs like COOH-MWCNTs are known to increase C_dl_ in modified electrode systems [46,47,51]. As such, it is extremely important to carefully construct these film assemblies with robust procedures and well-executed laboratory techniques to limit inevitable experiment-to-experiment variation, particularly with respect to the background signal.

The hypothesis of this study includes the concept that these modified electrodes allow XYL to traverse the PU layer via a diffusional rate that is largely dependent on the composition of that capping layer. After permeation through the PU layer, an applied oxidative potential would subsequently oxidize a limited sample of the XYL, with the by-product poised for subsequent reduction or “stripping” from the film, as the potential is swept negative during DPV. A critical aspect to understanding the role of the PU capping layer is that it redefines the diffusion layer at the electrode. The PU layer promotes electrochemistry of species within the layer. In the case of XYL, this occurs at the COOH-MWCNTs and GCE. In this respect, agitating the XYL solution to introduce mass transport by convection into the film did not result in enhanced signals (Supplementary Materials, Figure S9), suggesting the PU effectively “gates” XYL into the film as a function of primarily time and its inherent hydrophobicity. This type of entrapment behavior at PU-capped modified electrodes has been previously observed in other systems [51,57] and is particularly critical for understanding the results of this study. If proven effective, this adsorptive cathodic stripping method would utilize the same sensing interface as XYL oxidative sensors but provide and alternative for indirect XYL analysis that minimizes electrode fouling compared to other approaches [42,43,46,47].

### 3.3. Cathodic Reduction of XYL Oxidation Product—Potential Dependence

Electrochemical adsorptive stripping analyses typically involve a system that is responsive to the magnitude and duration of the oxidation potential applied before the cathodic sweep that generates the signal [61,62,63]. As observed from Figure 2B, the most prominent current response during a cathodic DPV scan (+1.4 → −0.8 V) applied to modified electrodes with either PU capping layer immersed in a XYL solution is the notably sharp peak at ~+0.35 V. The sharp peak is consistent with CNT-catalyzed reduction in the oxidized XYL, the idea being that application of positive potentials at the start of the cathodic sweep oxidizes a small portion of the XYL that has permeated the film, effectively sampling XYL from the bulk solution, a hypothesis that is reinforced with experiments described in subsequent sections. Prior to those experiments, a primary goal was to establish potential dependence of the cathodic signal.

**Figure 4:**
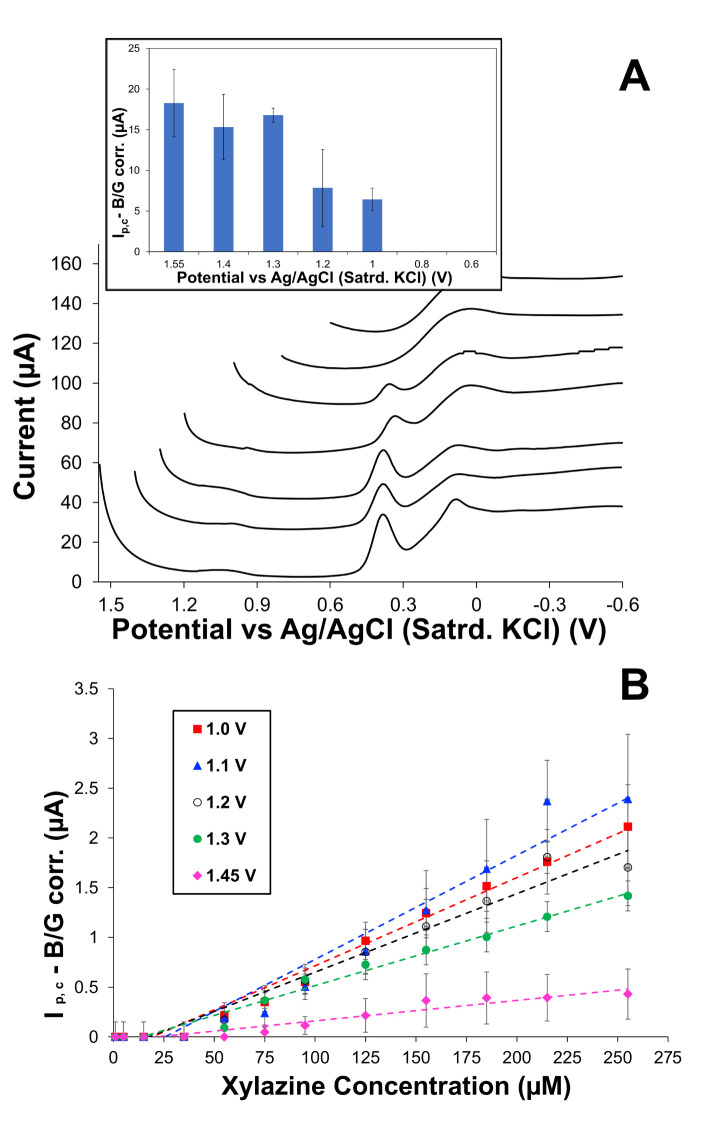
(**A**) Representative cathodic DPV scans of a 75:25 HPU–TPU-capped modified electrode in 1 mM XYL with different starting potentials (E_init_) and tracking of I_p,c_ as a function of E_init_ (inset). Note: DPVs are offset on the y axis for better visual interpretation of cathodic peak dependence on starting potential. (**B**) Calibration curves collected at different E_init_ showing sensor sensitivity as a function of E_init_. Note: Uncertainty is represented as standard error (n = 3).

If the initial potential (E_init_) allows for XYL oxidation and subsequently creates enough species for a detectable signal when it is reduced, it follows that there should be a potential dependence on the process (i.e., different E_init_ values should yield different cathodic peak currents (I_p,c_)). Figure 4A shows example DPV scans at different E_init_ values and establishes the potential dependence of the cathodic peak. As observed in the results, more positive E_init_ values yield larger I_p,c_, while some of the less positive E_init_ values resulted in no peak at all. Control experiments of cathodic DPV in XYL solutions with less positive E_init_ values produced voltammetry that essentially matches the same experiment in only PBS (no XYL) (Supplementary Materials, Figure S10). The potential dependence of the cathodic peak was robust, a result repeatedly achieved by multiple researchers at different times with numerous modified electrodes (Supplementary Materials, Figures S11–S13). Figure 4B expands upon this result by collecting calibration curves and current response versus increasing XYL concentration with the electrode poised at different E_init_ values. Notably, the calibration curves are all linear with respect to XYL concentration, regardless of E_init_, with sensitivity (i.e., slope) being similar for E_init_ of +1.0, +1.1, and +1.2 V. Calibration at the higher potentials (+1.3 and +1.45) results in lower slopes or dampened sensitivities, as it is suspected that the high applied oxidative potential may result in accelerated fouling of the electrode interface, while the lower potentials lessen that phenomenon [46,47]. Additionally, one of the stated goals of this project was to minimize high positive oxidation potentials, as it was common to see negative effects when oxidation was prolonged and repeated, performed on high XYL concentrations (mM), or with larger applied oxidating potentials. This was a critical finding in this study, and much of this project was typically conducted with E_init_ of +1.1 V for the cathodic DPV sweep in lower XYL concentrations and with careful attention to the duration of that applied potential.

### 3.4. Adsorptive Nature of the Modified Electrode System—Time Dependence

Adsorptive stripping voltammetry, in addition to having potential dependence, can also exhibit a time dependence most often associated with traditional stripping preconcentration or accumulation step that holds a potential over time for an initial deposition of material. For the system being investigated here, stripping mode was used to apply an initial oxidation potential for varying amounts of time prior to the cathodic DPV sweep. While modest gains in sensitivity could be observed with short deposition times of ~50 s (Supplementary Materials, Figures S14 and S15) from newly modified electrodes, the sensors did not benefit in sensitivity during calibration of the electrodes (Supplementary Materials, Figure S16), and there was no statistically significant increase in sensitivity when an oxidative deposition time was applied before each DPV sweep. These results suggest that, unlike Hg drop electrodes that can absorb electrolyzed species [64] or nanoporous substrates with significantly larger surface areas [65], the system investigated here behaves as a thin film more dependent on the time it takes for species to undergo diffusional adsorption through the PU layering. It also suggests that both the XYL and its oxidative products, the reduction of which is observed, are freely diffusing species within the film.

**Figure 5:**
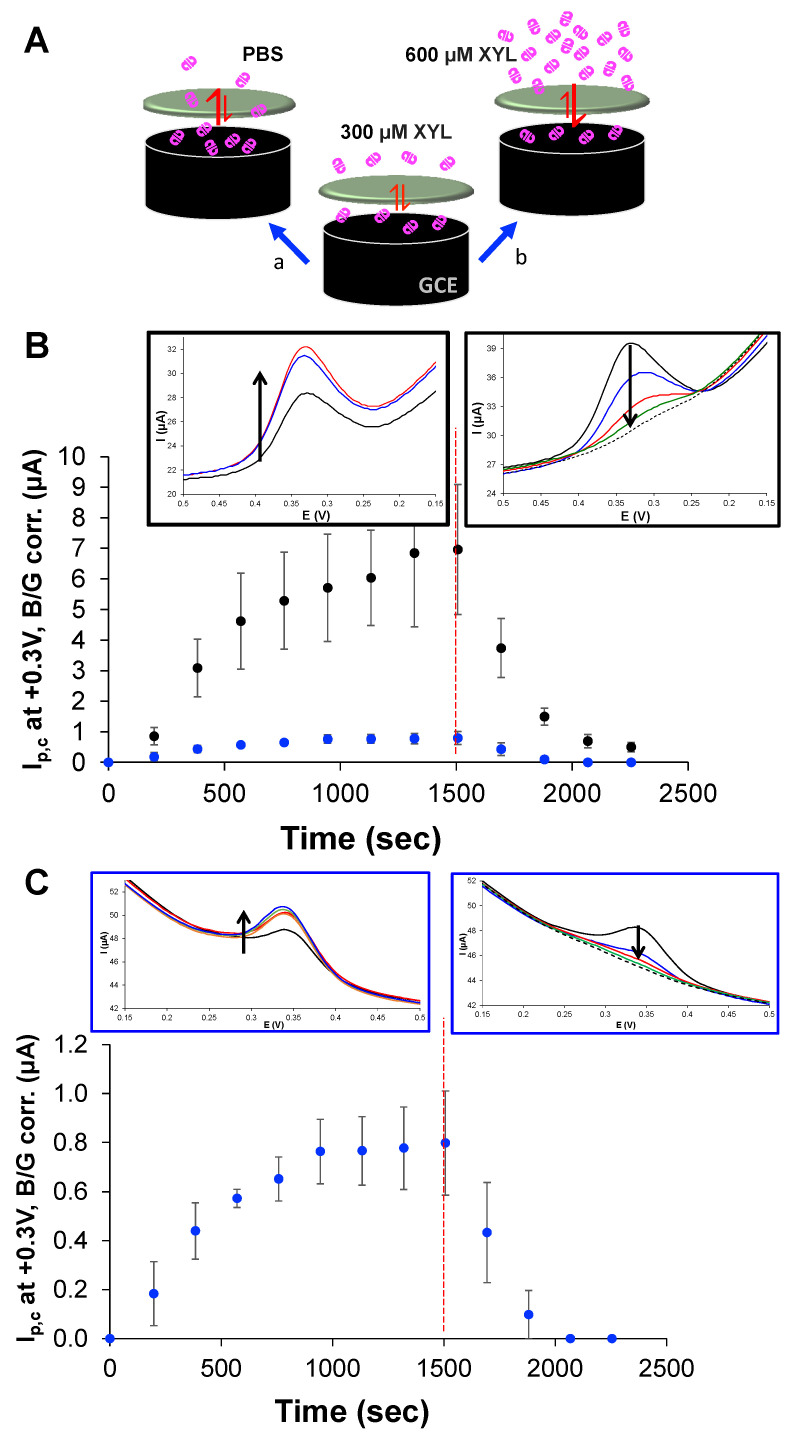
(**A**) Schematic representation of fully modified electrodes coming to equilibrium in 300 μM XYL before (a) being transferred to PBS or (b) to a more concentrated XYL solution (600 μM). (**B**) Tracking of i_p,c_ (corrected for background) from DPV scans (insets show examples) of both 75:25 (black) and 0:100 (blue) HPU–TPU-capped modified electrodes in 300 μM XYL solution as a function of time before being transferred (1500 s; - - - -) to a solution of PBS (0 μM XYL) and repeatedly scanned again. (**C**) Expansion of the results from (**B**) for the 0:100 HPU–TPU-capped system that show the same trend more clearly. Note: Uncertainty is represented as standard error.

To further establish the adsorptive nature of the modified electrode, specifically the role of the PU capping layer as a semi-permeable membrane, a series of strategic experiments were conducted. Effectively, the PU redefines the diffusion layer for that species within the film on the electrode [51]. In order to study this behavior, fully modified electrodes featuring both PU capping layers were immersed in a XYL solution (300 μM) to allow for interfacial equilibrium (i.e., XYL adsorption through the PU layers)—Figure 5A—Scheme. In these experiments, rather than waiting the standard 3 min (see Section 2) before applying potential to the systems, DPV sweeps were instead immediate and repeated after XYL injection and monitored over time to observe this initial equilibrium (Figure 5B,C). As the results show, repeated scanning results in a consistent cathodic peak growing with each scan regardless of the capping layer blend (Figure 5B,C—left insets) and eventually plateaus. At that point (1500 s), the modified electrodes are transferred to a PBS (0 mM XYL) and repeated DPV scans are again systematically collected over time. The results clearly show that the signal in the PBS diminishes over time with each scan until the signal disappears (Figure 5B,C—right insets). In examining these results, it is also clear that while the phenomenon is the same with both capping layers, the current flow with the HPU–TPU layer is larger. Similar experiments conducted on these systems, allowing for the 3 min rest time after the initial mixing, showed the same trends with the offset time, with both peak area and peak current tracking each other. These results are summarized in the Supplementary Materials (Figures S17 and S18).

An analogous experiment of this nature in the opposite direction was also conducted. A system with a 0:100 HPU–TPU capping layer was allowed to come to a stable signal in 300 μM XYL, before being transferred to a solution of twice that concentration (600 μM XYL) to monitor the cathodic signal as the system adjusted to the higher XYL concentration. Sequential and repeated cathodic DPV scans were conducted with and without the 3 min rest period, and both experiments showed expected responsiveness of increased current that eventually plateaued (Supplementary Materials, Figure S19). Collectively, this set of results suggest the PU layers behave as a gate-keeping membrane that, if given enough time to establish equilibrium with the XYL solution, regulate both the intake of XYL, the reduction in its oxidation product, and the exit of species from the film. The responsiveness of the systems to XYL concentration changes suggest that the cathodic DPV, starting at a potential to oxidize XYL, produces a detectable cathodic signal that could be used in quantitative analysis.

### 3.5. Adsorptive Cathodic Stripping Quantitative Analysis of XYL

**Figure 6:**
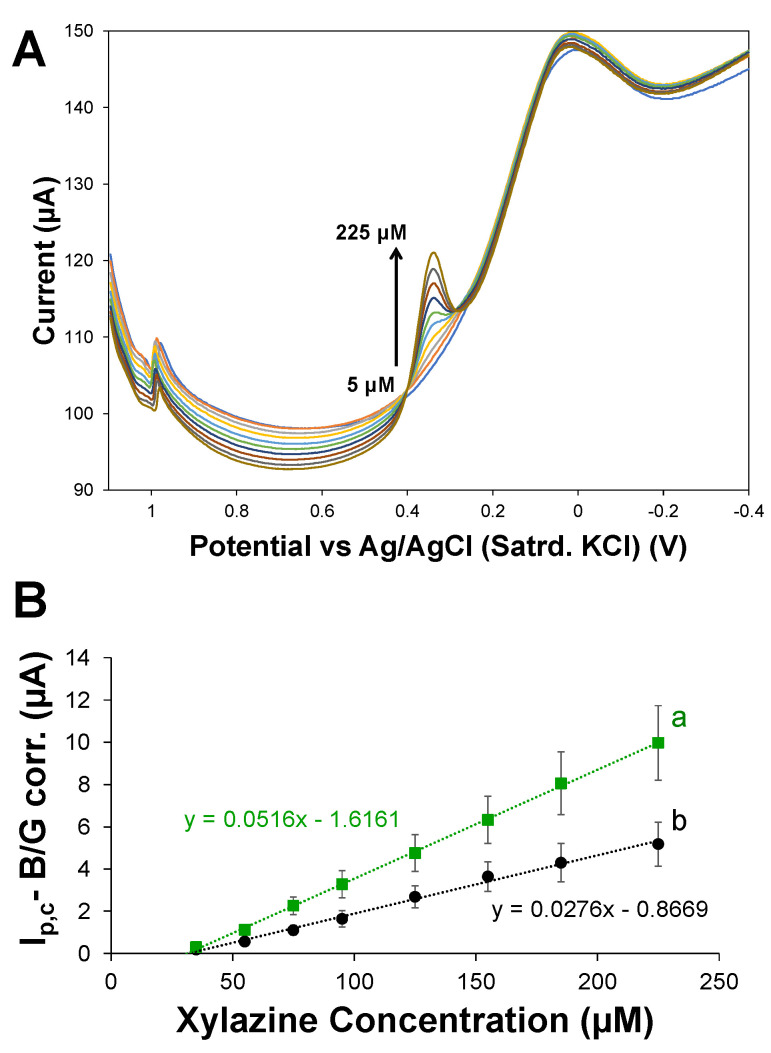
(**A**) Examples of overlayed cathodic DPV scans collected at the modified GCE utilizing the 75:25 HPU–TPU capping layer in increasing concentrations of XYL standard (5 to 225 μM XYL in 150 mM PBS at pH = 7) and (**B**) corresponding calibration curves created from background corrected I_p,c_ values for films capped with (a) 75:25 and (b) 0:100 HPU–TPU layers. Note: Uncertainty is represented as standard error.

This specific modified electrode scheme demonstrates an ability to sample XYL from a bulk solution and indirectly signal XYL via the reduction in an oxidation product. The limited exposure to a high oxidative potential for long periods of time is advantageous for expanding the scheme’s application for quantitative analysis of XYL. To demonstrate this capability, modified electrodes utilizing the 75:25 HPU–TPU capping layer were calibrated by running cathodic DPV analysis with E_init_ of +1.1 V after successive increases (i.e., injections) of XYL standard. Corresponding DPV scans, shown in Figure 6A, illustrate that the increasing I_p,c_ for the peak representing the reduction in the XYL oxidation product can be directly translated into a linear calibration curve (Figure 6B). An analogous set of experiments using the 100% TPU resulted in a linear calibration curve (Figure 6B) with slightly less sensitivity (i.e., smaller slope). Examples of repeated experiments of this nature, with both types of capping layers, are provided in Supplementary Materials (Figures S20 and S21). The general trend observed was that HPU–TPU-capped sensors seemed to consistently produce a higher current flow, suggesting such films are better able to allow for more XYL permeation. Importantly, the modified electrodes showed minimal shifts in the peak potential of the reduction wave during the generation of the calibration curve. In contrast, a similar experiment conducted at a bare GCE results in substantial shifts in peak potential and an eventual attenuation of current due to XYL exposure, resulting in a non-linear relationship between concentration and current (Supplementary Materials, Figure S22). Comparing the results of modified and unmodified electrodes illustrates that a systematic response to increasing XYL concentration, absent significant peak potential shifts typically associated with electrode passivation from fouling, can be achieved even after substantial XYL exposure during the calibration process.

The standard analytical performance of these modified electrodes used in this manner for quantitative analysis of XYL was assessed in PBS and yielded near 100% percent recovery. A XYL-spiked PBS solution with 125 μM XYL was compared to the calibration curve and yielded precent recoveries for the 75:25 and 0:100 HPU–TPU capped electrodes of 103(±7)%_n=4_ and 100(±13)%_n=4_, respectively (Table 1). As previously mentioned, the two types of capping layers tested showed slightly different sensitivities (Figure 6B), and their respective limits of quantification (LOQ or LOD·3) [41,66] were conservatively estimated at just below 10 ppm, on par with other voltammetry-based techniques that have been reported, though they are based exclusively on XYL oxidation [2,9,46,47]. While the analytical performance of using these sensors in this manner is reasonable in terms of those traditional evaluation parameters, the primary advantage of the cathodic-based approach is primarily the expansion of XYL detection to samples where oxidative approaches are problematic from background signals or overlapping interferent oxidation.

### 3.6. Application of the Sensor to Real Samples

**Figure 7:**
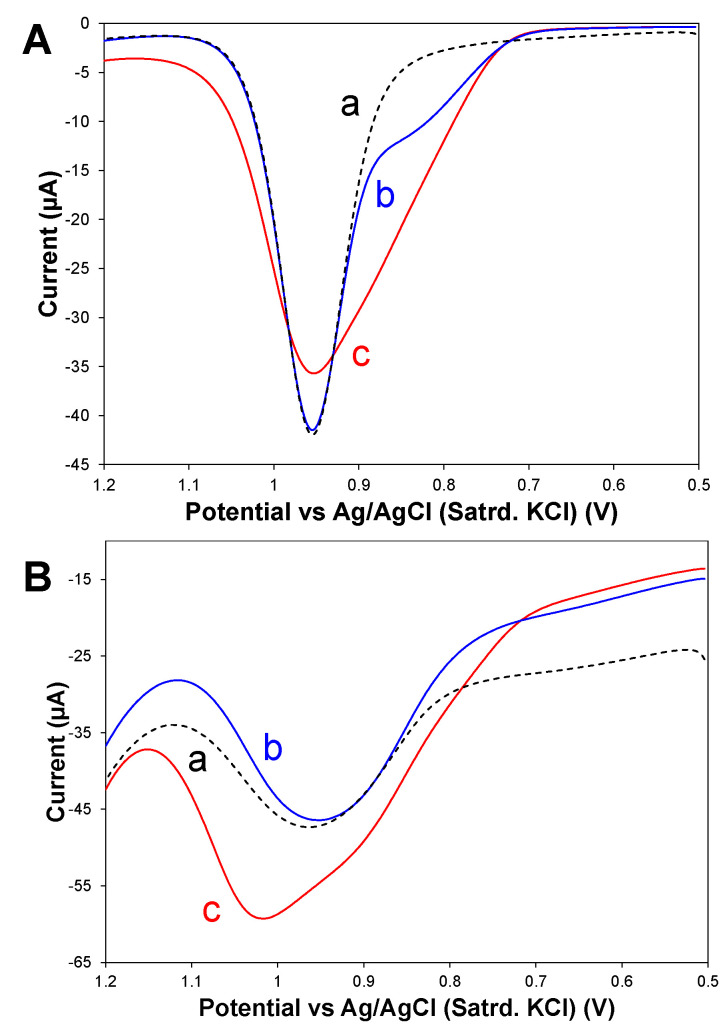
Anodic DPV scans collected at (**A**) bare and (**B**) fully modified (75:25 HPU–TPU) GCE electrodes immersed in (a) synthetic urine without XYL (dashed trace) and spiked with either (b) 125 μM or (c) 2 mM XYL showing oxidation peaks.

A sample medium of high interest for XYL detection is urine—a measurement that is impeded when using these modified electrodes for oxidative scans [46,47,48]. Figure 7 shows anodic DPVs of both bare and fully modified electrodes (75:25 HPU–TPU) during oxidative scanning in synthetic urine media with and without XYL spikes added. From these results, it is evident there are interferent species associated with the urine matrix, the oxidation of which overlaps and obscures the XYL oxidation signal, making this system an excellent example of where the adsorptive cathodic stripping developed in this study may be used as an alternative analysis method.

**Figure 8:**
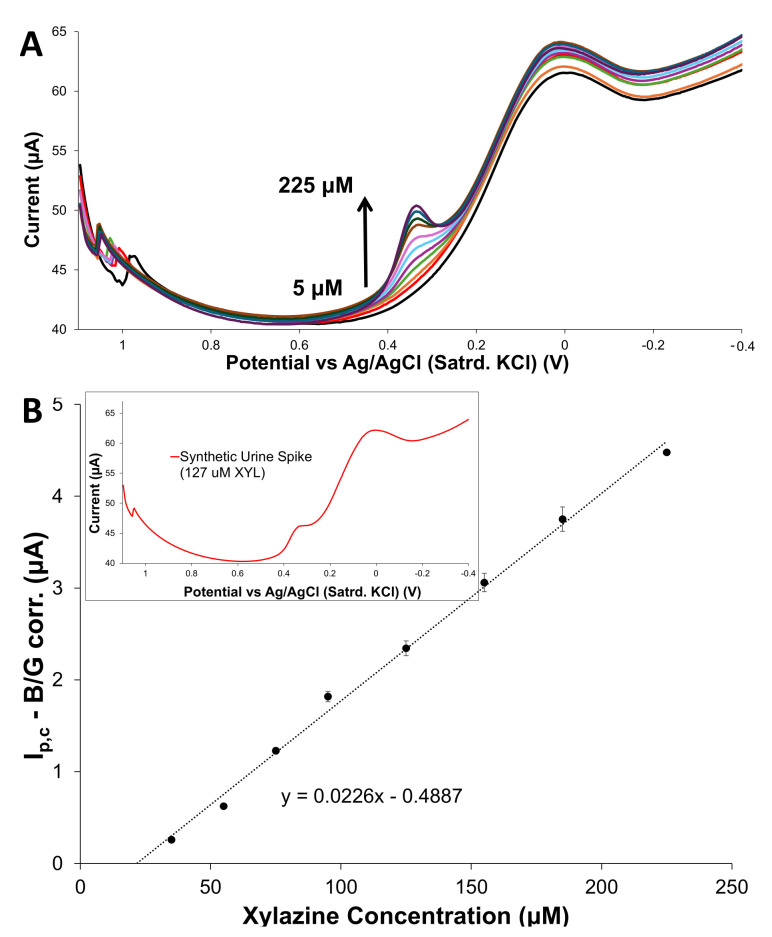
Example of XYL analysis of a real sample (synthetic urine) using adsorptive cathodic stripping including (**A**) cathodic DPVs collected in PBS during sequential increases in standard XYL concentration and (**B**) the corresponding calibration curve used to analyze the cathodic DPV signal from the spiked (127 μM) synthetic urine sample (inset). Note: In some cases, the uncertainty (standard error) is smaller than the markers.

For this analysis, calibration of fully modified electrodes (75:25 HPU–TPU capping layer) were used during sequential addition of XYL standard to a PBS solution and with DPV scans collected after each addition of the XYL (Figure 8). Background-corrected peak currents for the cathodic response were then used to create a calibration curve. The electrodes were then rinsed thoroughly before being immersed in a solution matrix of PBS, with the addition of a 500 μL aliquot of synthetic urine without XYL. After allowing the electrodes to become equilibrated in this solution, they were transferred to a new solution of PBS, this time spiked with 500 μL aliquot of synthetic urine containing ~36 mg of XYL (6.5 mM XYL). The resulting sample with a XYL concentration of 127 μM was analyzed using the cathodic DPV methodology and yielded a percent recovery of 105(±7)%_n=7_ (Table 1).

In addition to XYL detection in simulated urine, prior study also identified certain beverages that were unable to be analyzed using these sensors in an oxidative mode. Preliminary screening of sequential oxidative and reductive DPV scans for a select set of beverages (and synthetic urine) at unmodified GCEs are provided in Supplementary Materials (Figure S22). Representative samples of those beverages were analyzed using the adsorptive cathodic stripping method developed. To perform this analysis, new bottles of alcoholic beverages were purchased, sampled, and spiked with XYL before an aliquot of the beverage was diluted in a vessel of freshly prepared PBS so that the XYL concentration was 127 μM. These experiments were again largely successful at quantifying XYL from samples of those spiked beverages. The collective results from adsorptive stripping analysis testing of XYL-spiked real samples, beverages, and synthetic urine, are summarized in Table 1. Overall, the results suggest that the blended PU capped sensors (i.e., HPU–TPU) performed better in the different media. As proof-of-concept, both types of sensors showed successful detection of XYL in select beverages found to be problematic in previous oxidation-based analyses [46,47], including XYL in samples of whiskey and hard seltzer. For comparison, a recently published spectroscopic method for XYL detection using dye displacement from molecularly imprinted polymer substrates [40] showed successful quantification, with an LOD of 1.36 mM, but was also pH dependent, reliant on the percent volume of alcohol in the system; the sensor was only tested on one beverage matrix (gin and tonic).

### 3.7. Potential Mechanism of XYL Redox Chemistry

**Figure 9:**
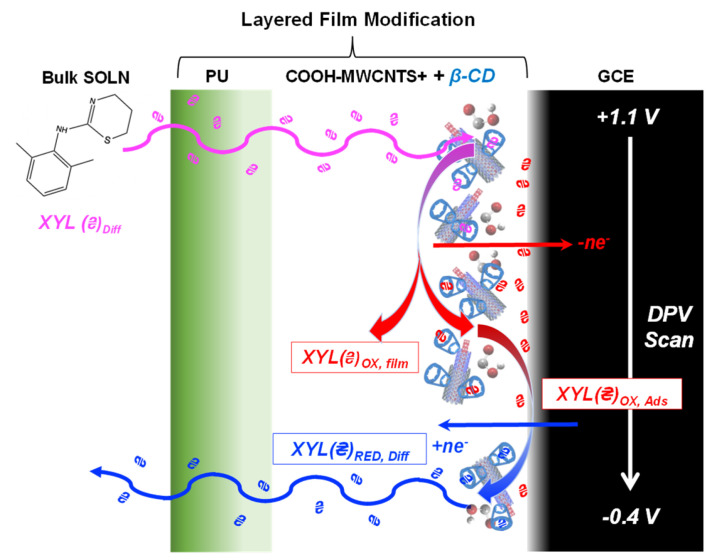
Schematic of proposed electrochemical mechanism for adsorption cathodic stripping experiment of XYL analysis showing initial permeation of XYL through the film where it is subsequently oxidized during the early stages of the cathodic sweep (pink arrow) before that oxidized product either is adsorbed to the electrode as a fouling agent or remains suspended within the film (red arrows) and available for reduction of that species as the applied potential sweeps negative (blue arrow) and subsequently that species exits the film.

While not the focus of our study, the collective results of this study regarding XYL redox activity within the film layer suggest an electrochemical mechanism most consistent with the detailed redox chemistry proposed by Mendes et al. at a bare GCE [48]. Though not definitive, the results within this study do suggest that the oxidative electrolysis at the amine site of XYL results in the consumption of XYL and the production of two species, one prone to adsorption at the electrode interface. This adsorbed species is identified in the Mendes report. Our study expands upon that adsorbed species, suggesting there are other oxidative products, perhaps oxidized at the modified MWCNTs, that remain diffusional within the film rather than fouling the electrode. The diffusional oxidized species is then available for reduction during cathodic sweeps. Our best attempt at modeling this complex film and its associated electrochemistry is illustrated in Figure 9. The exact role of the adsorbed oxidation product in subsequent sensing remains unclear. Regardless of the mechanism, demonstration of both direct and indirect detection of XYL suggests an avenue for research in sensor development targeting other nefariously employed/abused pharmaceuticals.

## 4. Conclusions

This study accomplished the goals of both a better understanding of the modified electrodes and the role of the semi-permeable PU layers, as well as establishing that the cathodic reduction in the XYL oxidation products within that film can be used as an indirect signal for the quantitative analysis of XYL. In particular, the “gating” of XYL through the PU outer layer, previously identified in other studies as critical for both selectivity considerations as well as inter-film electrochemistry [46,47,51], is clearly established by this work. In demonstrating that the same type of modified electrodes, utilizing the same materials, can detect XYL in both an oxidative and reductive mode, the range of samples that can be investigated for containing XYL is expanded - a significant finding of the work. Additionally, based on literature reports, it is likely that this proof-of-concept study can be further optimized via additional adjustment of DPV-specific parameters [42], further adjustment of the PU properties, and/or employing standard additional quantitative analysis [46] for matrices that, while not specifically explored here, may still have persistent interferent issues. Importantly, the functionality of the XYL detection is demonstrated to be effective down to 10 ppm or, for example, a 1.5–2 mg spike of XYL into a 5 oz (~150 mL) drink—all well below the reported toxicity levels for humans [38]. Thus, in addition to the versatility of cathodic measurement, the sensitivity of the sensor is appropriate for detecting XYL in a drink both prior to consumption (XYL likely in mg or g per mL) for crime prevention as well as post-consumption (e.g., residual or diluted from melted ice), as would be needed for investigation of sexual assault [10]. Development of such tools can help combat the rapidly emerging threats of using such compounds as adulterants in narcotics [2] and, in the case of XYL, its continued illegal use in facilitating sexual assaults [39], two unfortunate applications of XYL that afflict society at an unacceptable rate [7,9].

## Figures and Tables

**Table 1:** Quantitative analysis of XYL in real samples (% recoveries).

Sample	PU Layer	Volume Sampled (µL)	Dilution in 150 mM PBS (mL)	Spike [XYL] (µM)	Trials (n)	Avg % Recovery
PBS (150 mM)	HPU–TPU	62.5	25.06	125	4	103 (±7)
PBS (150 mM)	TPU	62.5	25.06	125	4	100 (±13)
Synthetic Urine	HPU–TPU	500	25.5	127	7	105 (±7)
Synthetic Urine	TPU	500	25.5	127	4	130 (±11)
Whiskey	HPU–TPU	500	25.5	127	4	76 (±12)
Whiskey	TPU	500	25.5	127	3	69 (±6)
Hard Seltzer (Peach)	HPU–TPU	500	25.5	127	3	107 (±24)
Hard Seltzer (Peach)	TPU	500	25.5	127	4	104 (±10)

Notes: Samples are PBS = phosphate buffer solution (150 mM; pH 7); synthetic urine = Sigmatrix Urine Diluent (Oakwood Chemical); whiskey = Wild Turkey; peach hard seltzer = Whiteclaw; uncertainty is represented as standard error.

## Data Availability

The data that support the findings of this study are available from the corresponding author upon reasonable request.

## Supplementary Materials

The following supporting information can be downloaded at: https://www.mdpi.com/article/10.3390/s25175312/s1, Figures S1: Electron microscopy imaging of bare and modified substrates; Figure S2: Amperometry of electrodes in XYL; Figures S3–S6: Cyclic voltammetry of K3Fe(CN)6 and XYL at various interfaces; Figures S7–S13: Cathodic DPV scans at various interfaces, solutions, and DPV parameters (e.g., start potential); Figures S14–S16: Analysis of oxidative deposition time effects; Figures S17–S19: Tracking peak current and area as a function of time with DPV at various films; Figures S20 and S21: DPV overlays and corresponding calibration curve analysis for films of both capping layers; Figure S22: DPV of increasing XYL concentration at a bare GCE; Figure S23: Oxidative and reductive DPV scans of bare GCE in various sample media.
