# Supplementary material for: Adsorptive Cathodic Stripping Analysis of Xylazine Within Fouling-Resistant and Nanomaterial-Enhanced Modified Electrode Sensors
Source: Sensors (Basel). 2025 Aug 26;25(17):5312. doi: 10.3390/s25175312 (PMC12431543; doi:10.3390/s25175312)
Supplement: Supplementary file 1 [file sensors-25-05312-s001.zip › sensors-3808933-supplementary.pdf]

Supplementary Materials:

Adsorptive Cathodic Stripping Analysis of Xylazine within Fouling-Resistant and Nanomaterial-Enhanced Modified Electrode Sensors

Michael C. Leopold,* Charles W. Sheppard, Joyce E. Stern, Arielle Vinnikov, Ann H. Wemple, and Ben H. Edelman

**Department of Chemistry, Gottwald Center for the Sciences, University of Richmond, Richmond, Virginia 23173, United States*

Table of Contents:

▪ Electron microscopy imaging of dispersed MWCNT/β-CD (TEM) and SEM imaging of bare/modified GCEs (**Figs. S1**)

▪ Amperometric I-t curves showing bare and modified electrode XYL exposures (**Fig. S2**).

▪ Cyclic voltammetry of unmodified GCE in 1 mM XYL solution at different scan rates with peak current tracking per cycle (**Fig. S3**).

▪ Cyclic voltammetry of GCE in 5 mM FeCN solution after previous exposure of GCEs to cyclic voltammetry at different scan rates in 1 mM XYL solution (**Fig. S4**).

▪ Cyclic voltammetry of unmodified and fully modified GCEs in 1 mM XYL solutions with corresponding background voltammetry (**Fig. S5**).

▪ Cyclic voltammetry overlay of unmodified and fully modified GCEs in 1 mM XYL solutions (no background voltammetry included) (**Fig. S6**).

▪ Cathodic DPV overlays of fully-modified electrodes capped with either 75:25 or 0:100 HPU:TPU layers in XYL solution after a 200 and 900 second wait (**Fig. S7**).

▪ Cathodic DPV overlays of various modified electrodes including fully-modified electrodes, fully-modified electrodes with significantly less CNTs, PU-capped GCE, and GCE (bare) (**Fig. S8**).

▪ Cathodic DPV scans with and without stirring for fully modified electrodes capped with either a HPU:TPU blend or 100% TPU layer **(Fig. S9).**

▪ Cathodic DPV scan overlay of fully modified GCEs in 1 mM XYL solutions with starting scan potentials (E_{init}) of +1.4 and +0.5 V (**Fig, S10**); with the latter starting potential repeated with and without XYL in at bare and modified GCE (**Fig. S11**).

▪ DPV overlay of fully-modified GCE in 1 mM XYL solutions with different starting potentials (E_{init}) (**Fig. S12**) and tracking of peak current (**Fig. S13**).

▪ Tracking of the cathodic reduction peaks area during DPV stripping mode scans of (A) 75:25 and (B) 0:100 HPU:TPU capped modified electrodes in 300 μM XYL (**Fig. S14**).

▪ Representative cathodic DPSVs at of fully modified GCEs immersed in 1 mM XYL (**Fig. S15**).

▪ Calibration curve created from cathodic DPV responses ($i_{p,c}$) with/without deposition holds (**Fig. S16**).

▪ Tracking of peak current and peak area for the two PU capping layers after transfer to PBS (**Fig. S17,18**) or a more concentrated XYL solution (**Fig. S19**).

▪ Additional examples of cathodic DPV scans collected at the modified GCE utilizing the 0:100 HPU:TPU (**Figure S20)** or 75:25 HPU:TPU capping layers (**Fig. S21**) in increasing concentrations of XYL standard and corresponding calibration curve; Similar DPV scans at a bare GCE showing fouling (**Fig. S22**).

▪ Preliminary oxidative and reductive scans of bare GCE in various beverage samples (**Fig S23**).

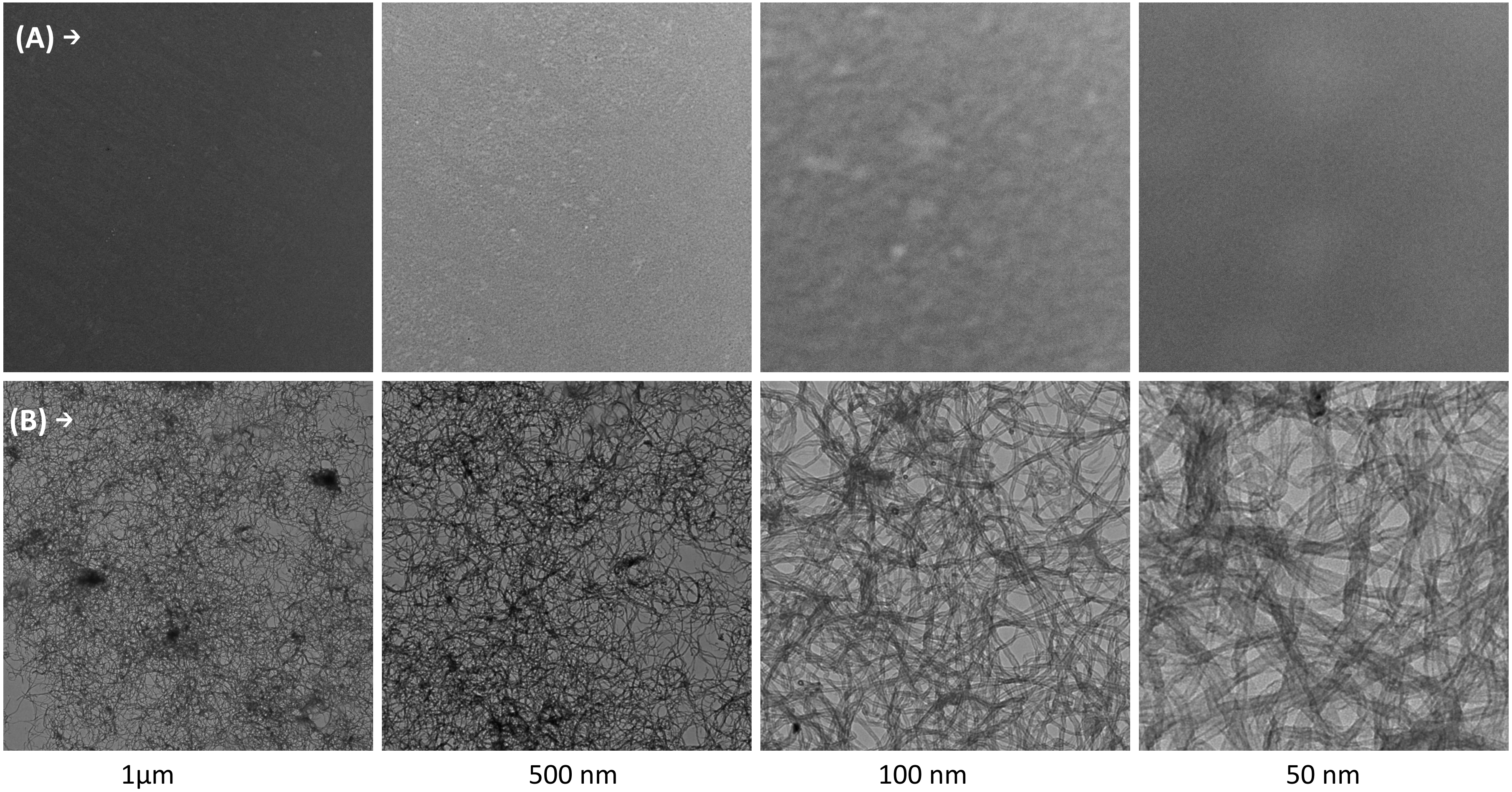

Figure S1: Transmission electron microscopy imaging of TEM grids **(A)** bare and **(B)** with dispersed COOH-MWCNTs with β-CD at different magnifications.

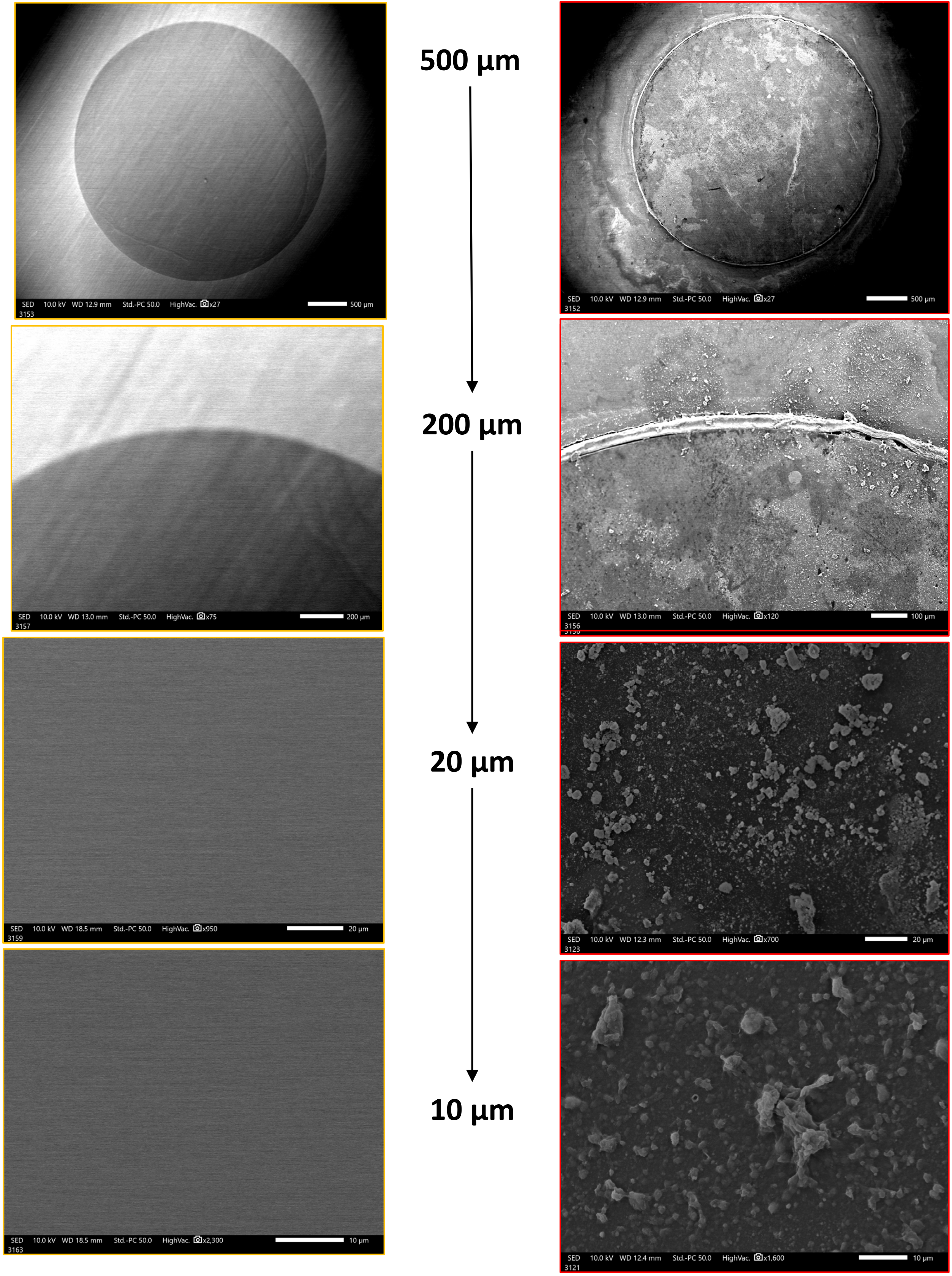

Figure S1: SEM imaging of a bare (*left*) and MWCNT/β-CD modified (*right*) GCEs at various magnifications (*center = white measure bar length, bottom right per image)* with a 1 µm scan shown on the next page. Note: These GCE are actual electrodes used in the electrochemistry cut to fit the SEM instrument.

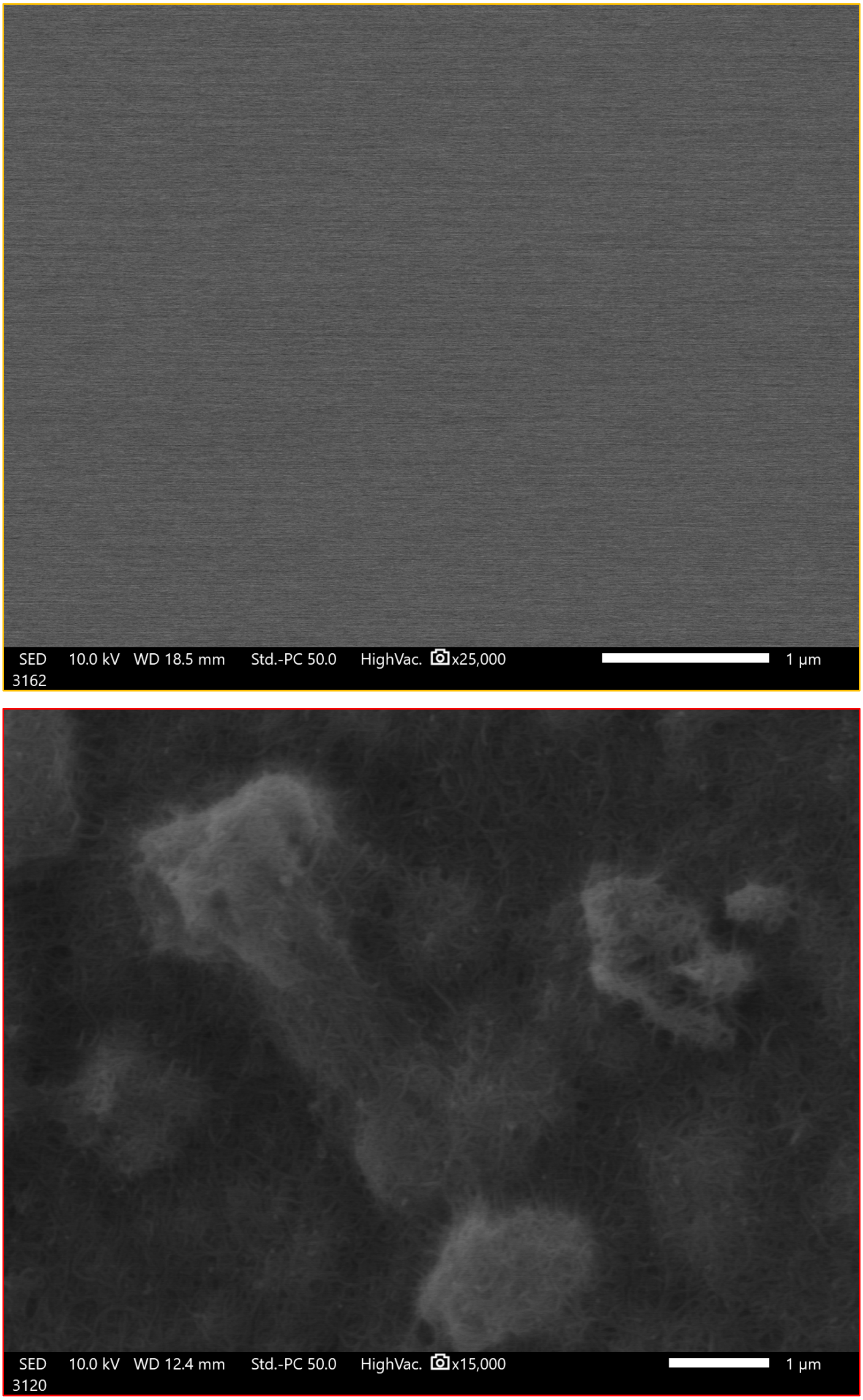

Figure S1: SEM imaging of a bare *(top)* and MWCNT/β-CD modified (*bottom*) of GCEs at high magnification (white bar = 1 µm). Note: These GCE are actual electrodes used in the electrochemistry cut to fit the SEM instrument.

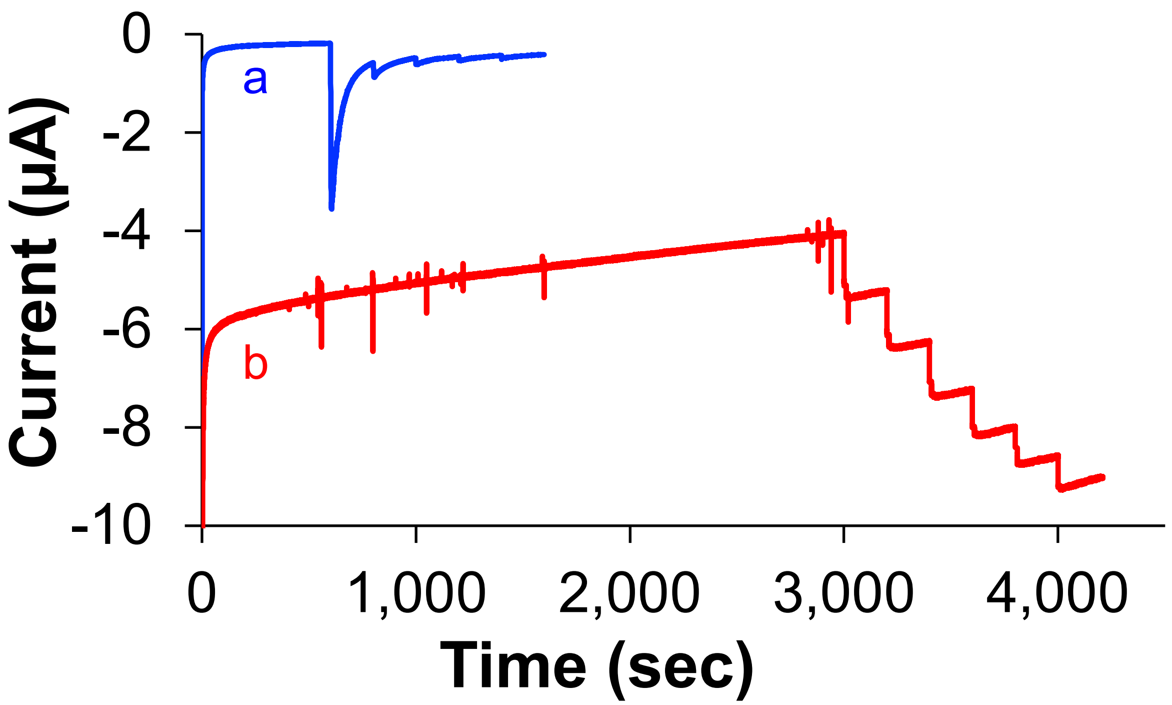

Figure S2. Examples of I-t curves of **(a)** bare and **(b)** fully-modified electrodes with a 75:25 HPU:TPU capping layer used to illustrate XYL exposure prior to results shown in Fig. 1. A +1.1V applied potential was held constant during successive 50 µL injections of 50 mM XYL standard ~ every 200 seconds. Notably, the response at the bare electrode (a) quickly diminishes over time as the electrode interface becomes fouled, in stark contrast to the response at the modified electrode (b).

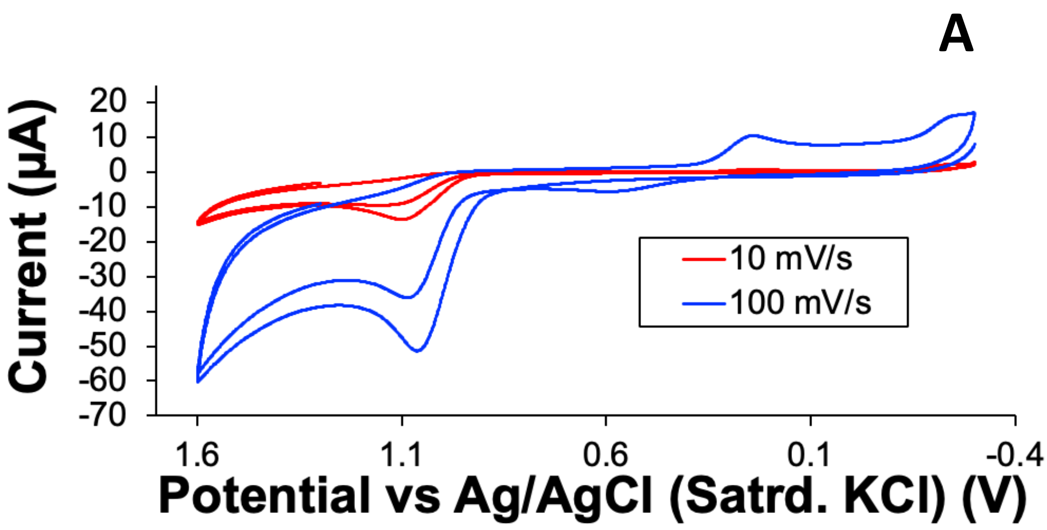

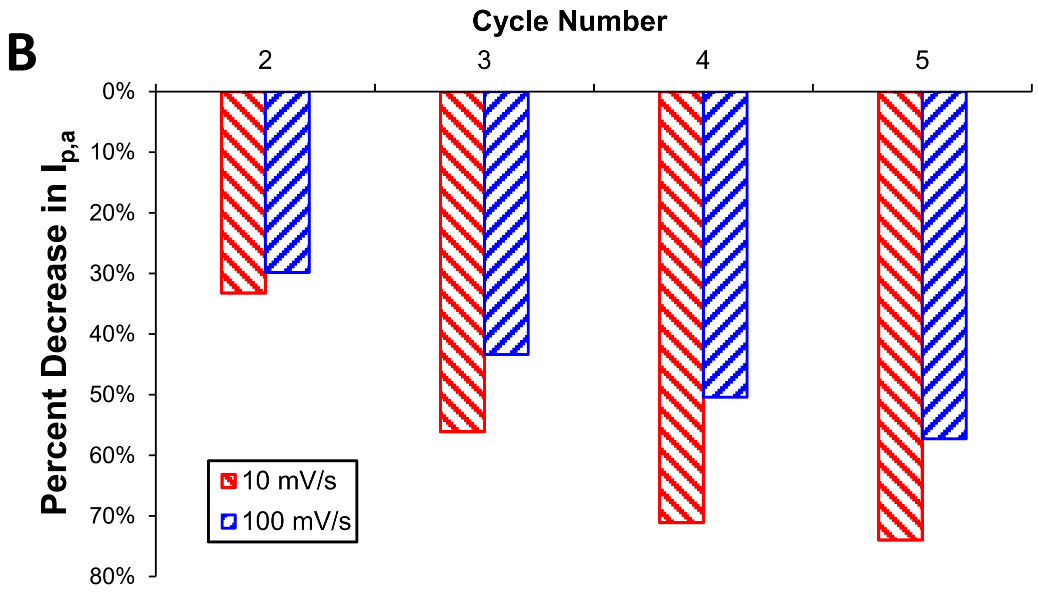

Figure S3. (A) Successive cyclic voltammetry (CV) scans of unmodified GCE electrode immersed in 1 mM XYL (150 mM PBS at pH =7) at **100 mV/sec** and 10 mV/sec scan rates; **(B)** Plot of the % current signal ($I_{p,a}$) for peak at ~+1.0 with each cycle/scan for the different scan rates.

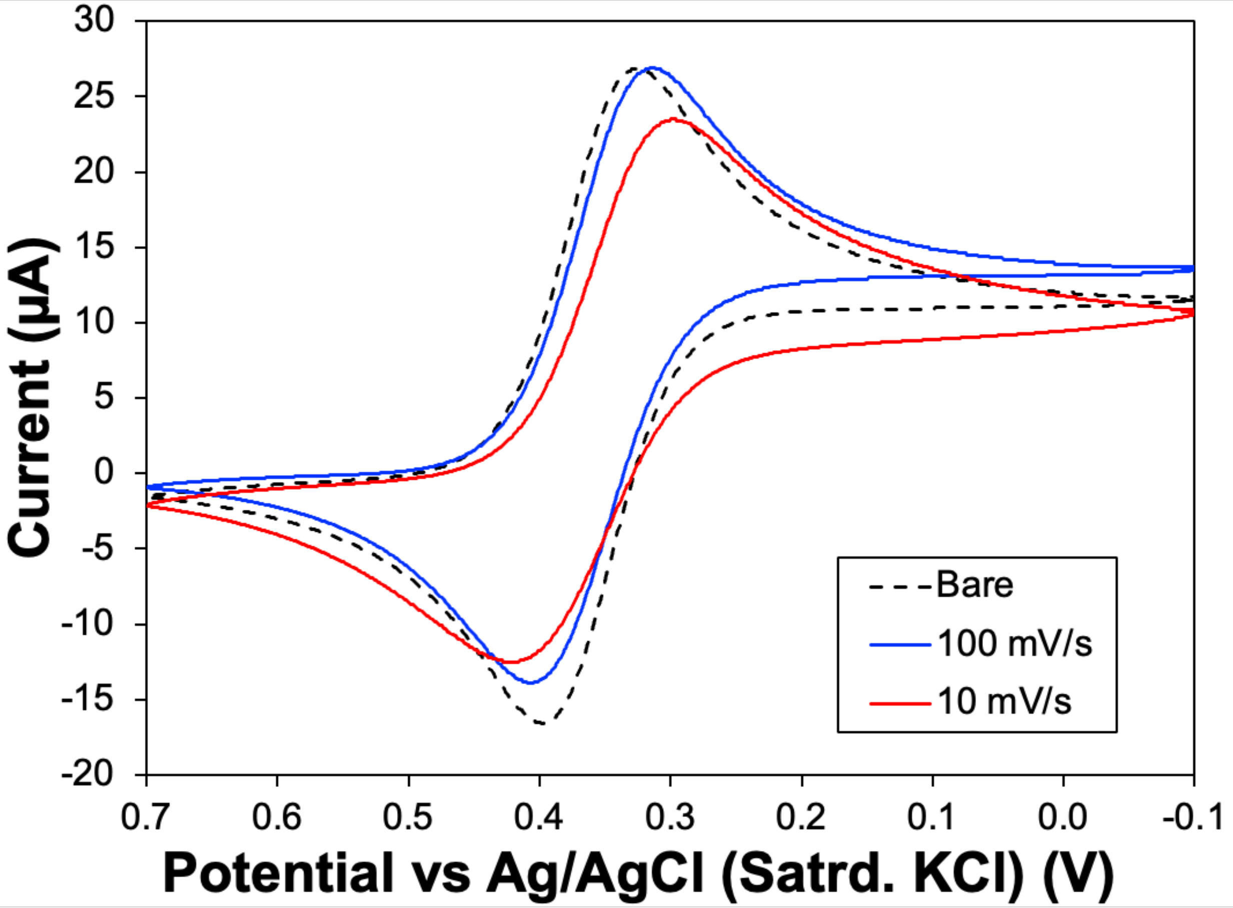

Figure S4. Representative CV of 5 mM potassium ferricyanide (FeCN) in 0.5 KCl at unmodified GCEs before and after XYL exposure of CV in 1 mM XYL at either 100 mV/sec or 10 mV/sec.

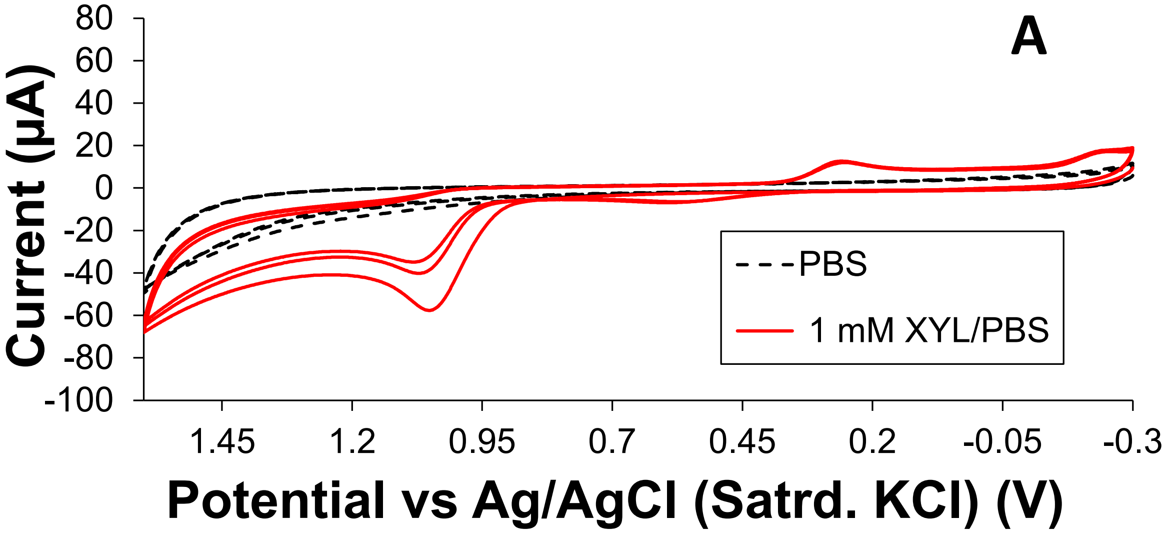

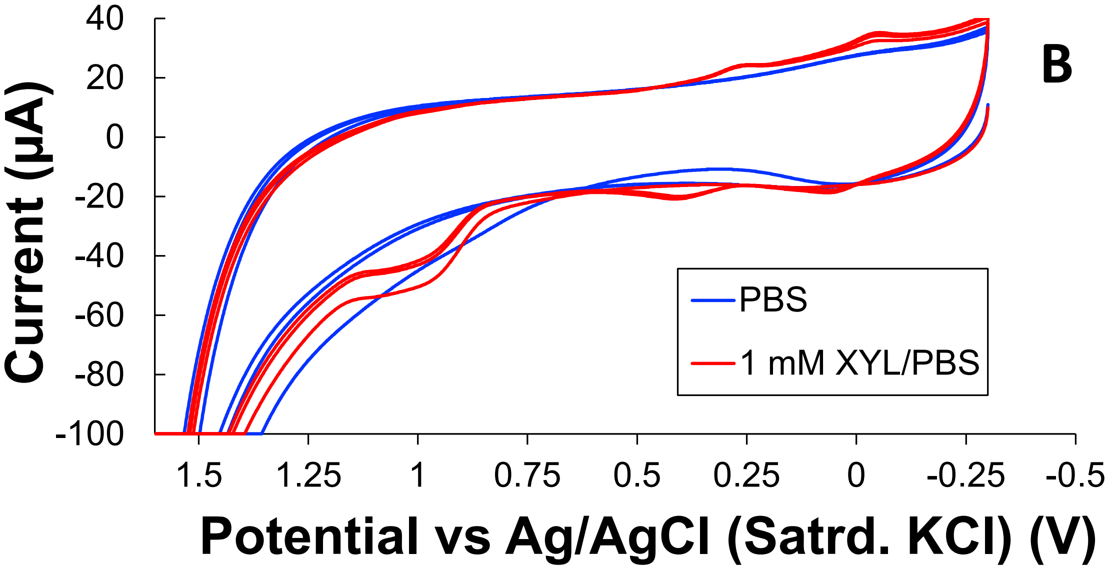

Figure S5. CV of **(A)** unmodified GCE and **(B)** fully modified (Scheme 1) GCE with 75:25 HPU:TPU capping layers in 1 mM XYL (150 mM PBS at pH =7) with corresponding background scans (3 cycles).

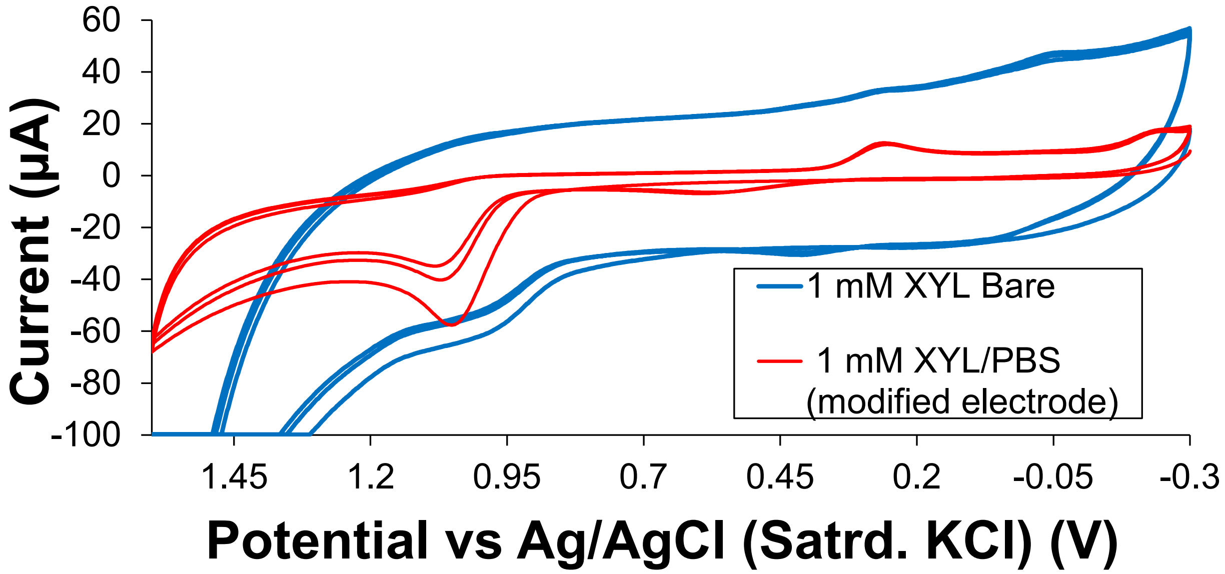

Figure S6. CV overlay of unmodified or bare GCE and fully modified (Fig. 2A) GCE with 75:25 HPU:TPU capping layers in 1 mM XYL (150 mM PBS at pH =7). Note: Background scans in PBS are excluded for visual clarity.

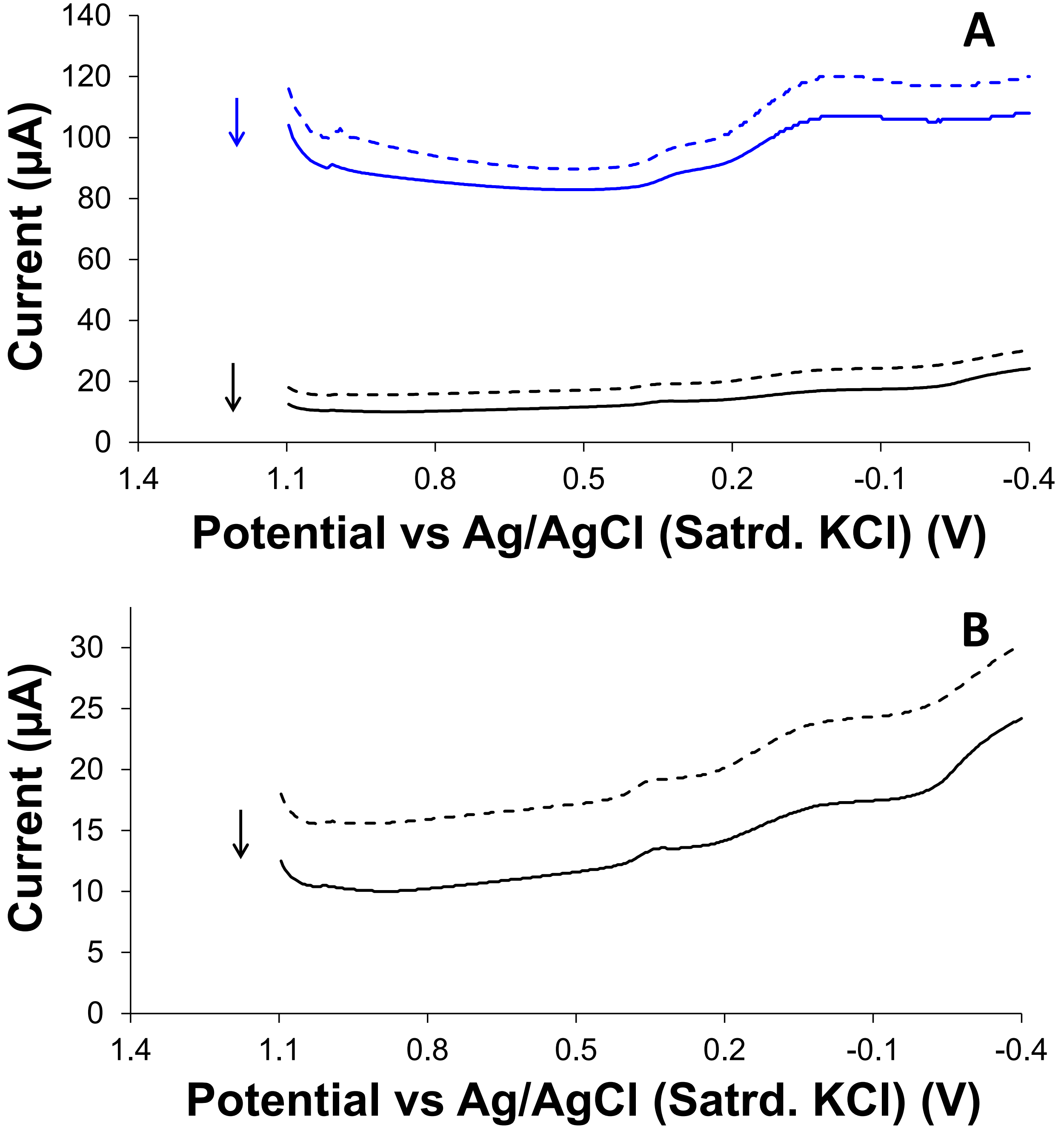

Figure S7. (A) Overlays of initial DPV scans of modified electrodes capped with either 75:25 (*blue*) or 0:100 (*black*) HPU:TPU capping layers after 200 second (*dashed traces*) or 900 second (*solid traces*) wait times in 300 µM XYL (150 mM PBS at pH =7) and; (B) expansion of the 100% TPU results for clarity.

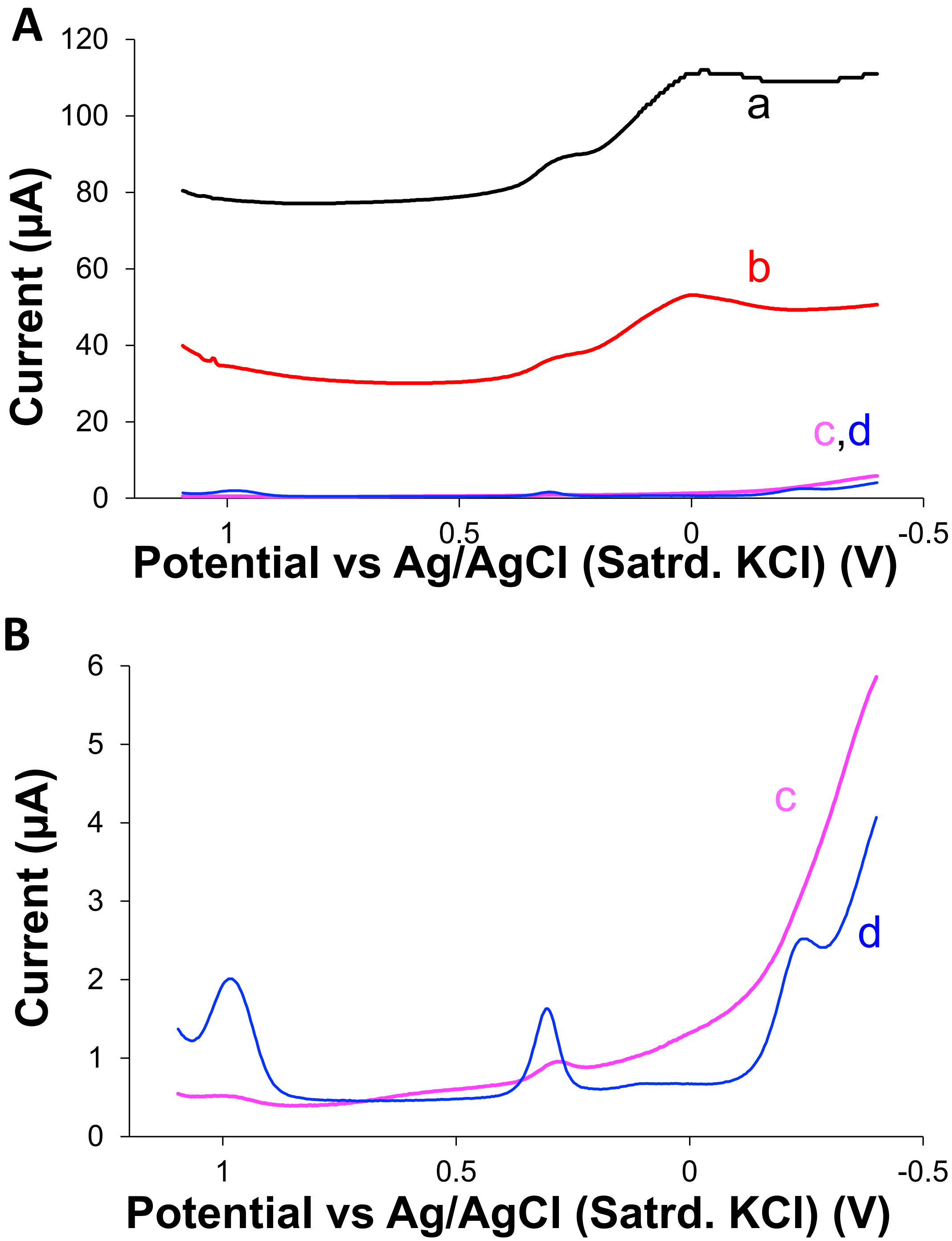

Figure S8. **(A)** DPV overlays (not offset) of various types of electrode modifications in 300 μM including: (a) fully-modified (b) partial film modification (less MWCNT coverage); (c) GCE modified with only PU layer (75:25 HPU:TPU) and; (d) clean, bare (unmodified) GCE; **(B)** Expansion of DPVs from (A). Notes: XYL mixture was stirred for 10 sec (1000 rpm) followed by 3-minute rest period; potential was held at +1.1V for 15 seconds preceding the DPV sweep in each case.

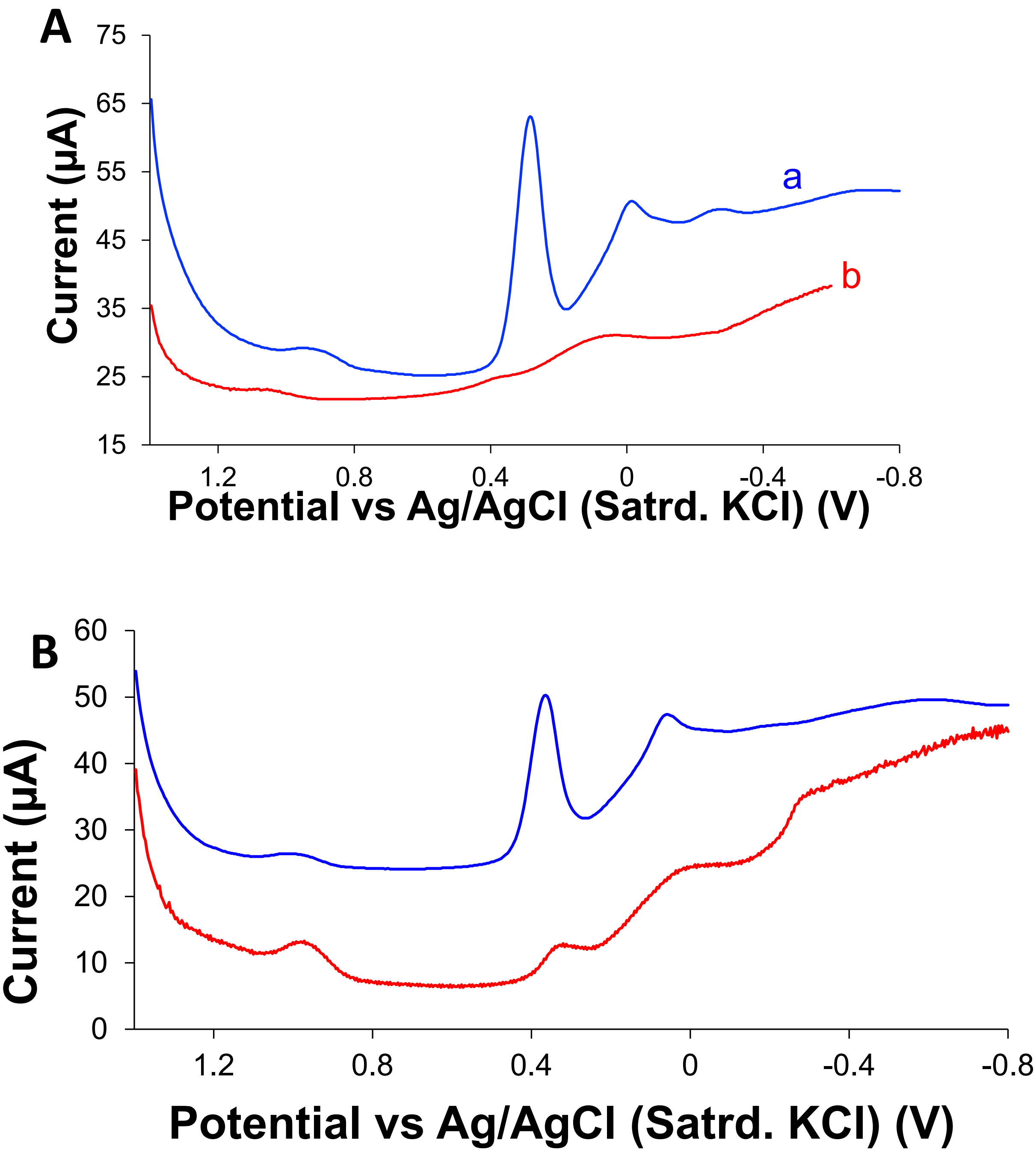

Figure S9. Cathodic DPVs of modified electrodes capped with **(A)** 75:25 or **(B)** 0:100 HPU:TPU layers in 1 mM XYL (150 mM PBS; pH =7) with (a) diffusional and (b) convection (stirring) mass transfer present during the DPV scan.

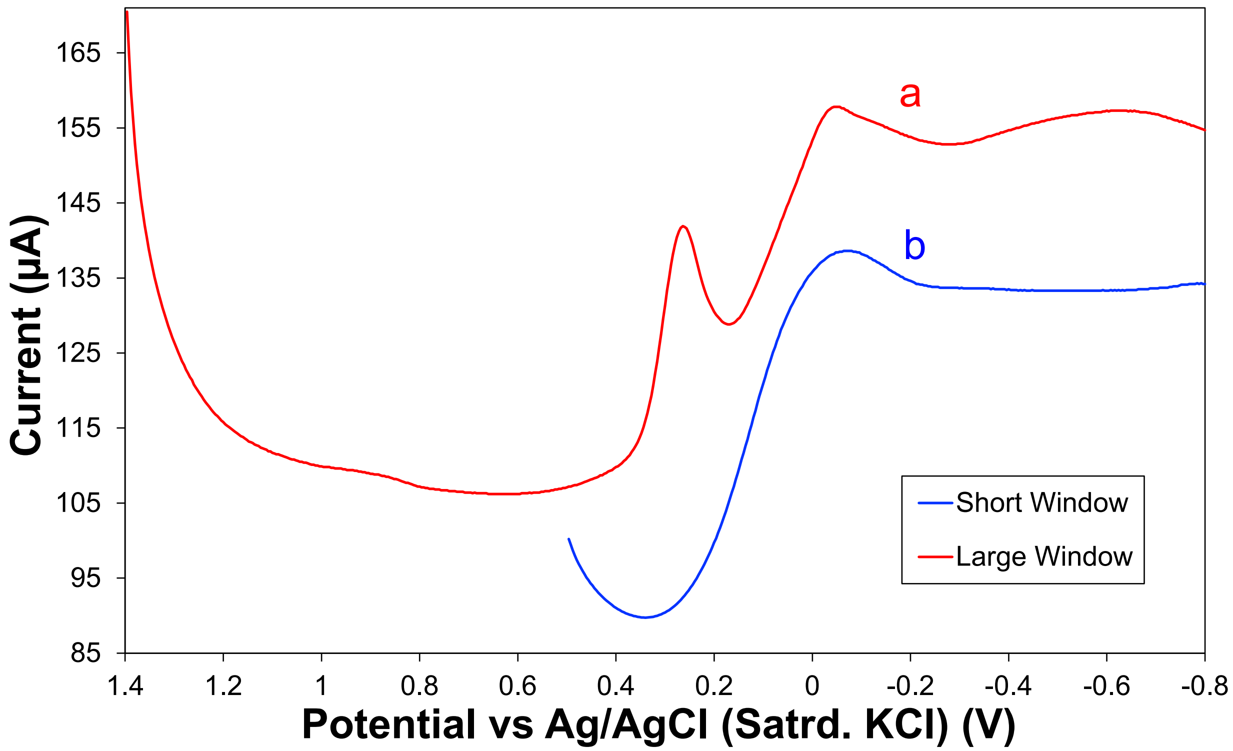

Figure S10. Cathodic DPV overlay of fully modified (Fig. 2A) GCEs in 1 mM XYL (150 mM PBS at pH =7) with a E_{init} of **(a)** +1.4 V and **(b)** +0.5 V.

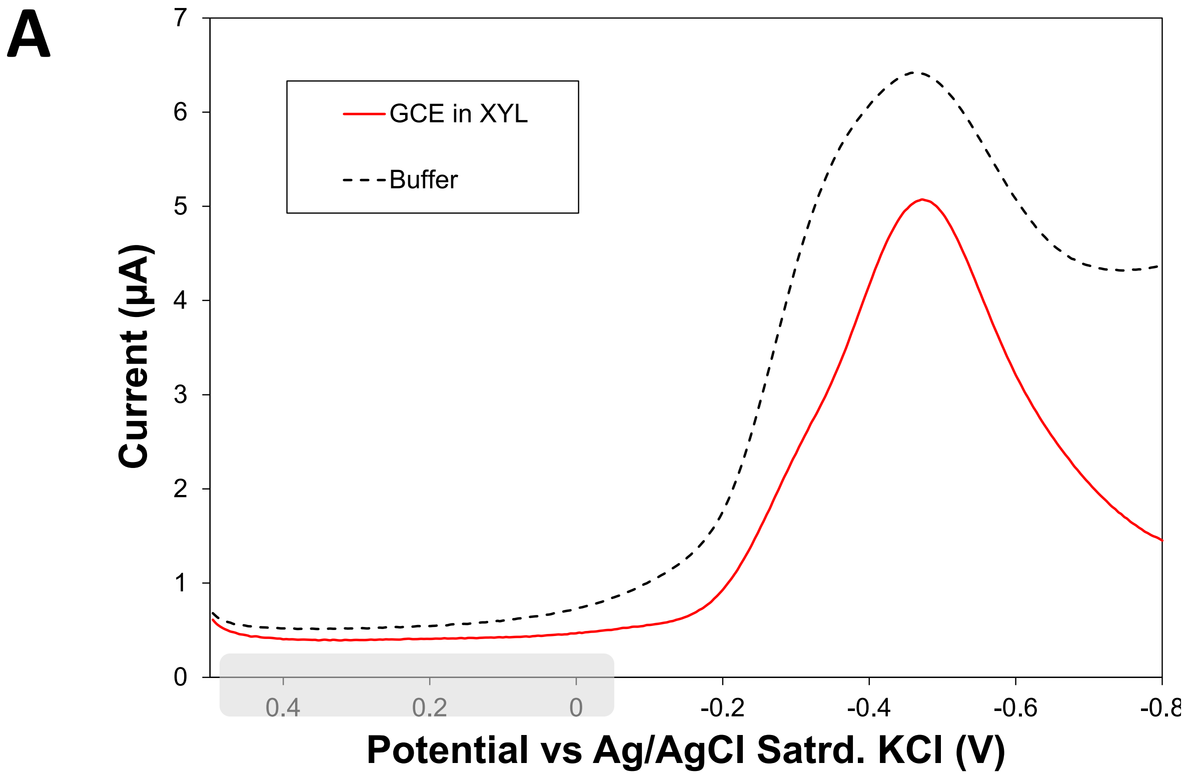

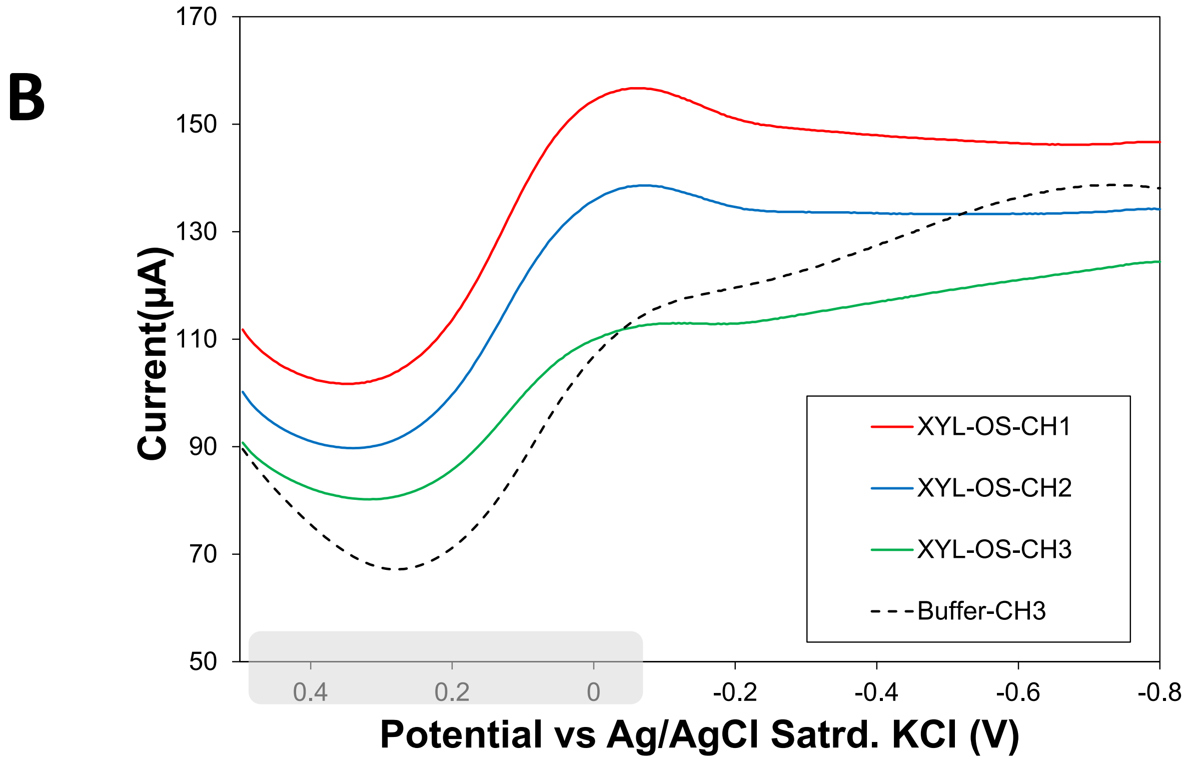

Figure S11. DPV overlays of **(A)** unmodified GCE and **(B)** three fully modified (Fig. 2A) GCEs with 75:25 HPU:TPU capping layers in 1 mM XYL (150 mM PBS at pH =7) vs. background in PBS (150 mM; pH =7), *dashed traces*. Note: Shaded area (*grey*) designates potential range where cathodic reduction peak for XYL is expected.

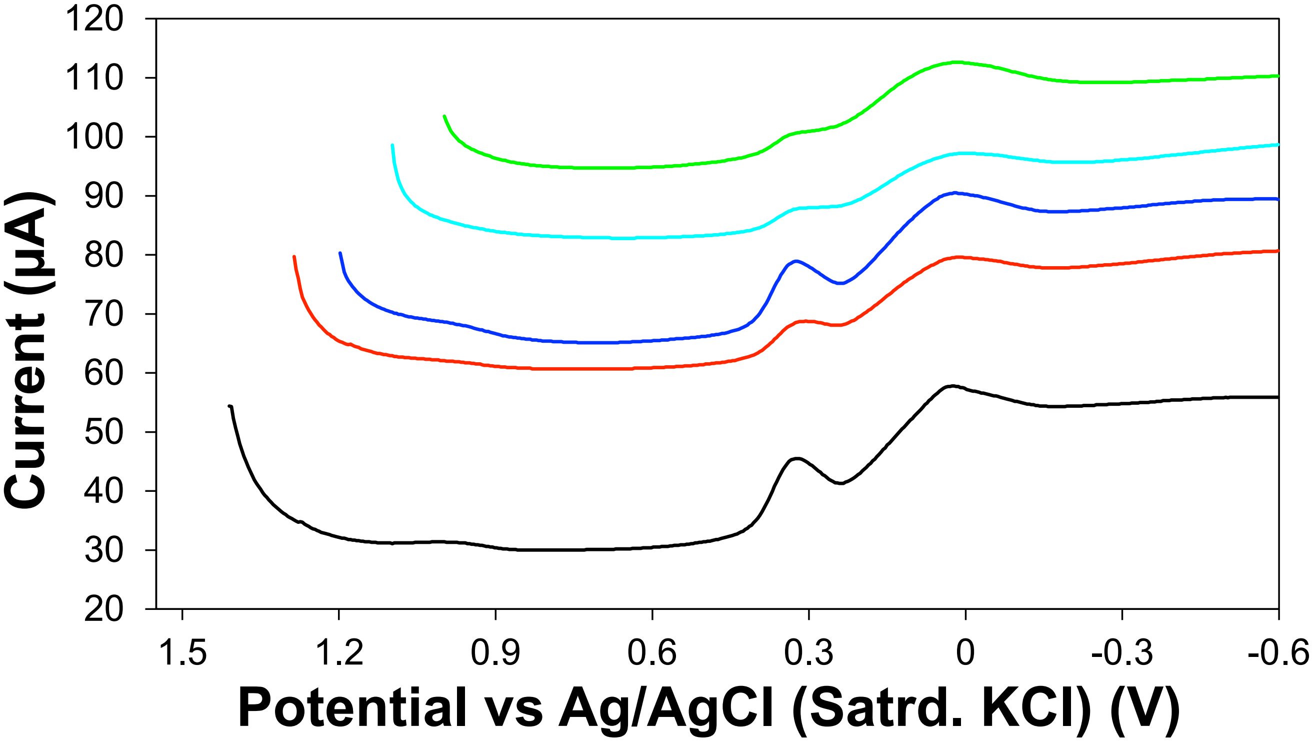

Figure S12. Cathodic DPV scans of a fully-modified (Fig. 2A) GCE with 75:25 HPU:TPU capping layers in 1 mM XYL (150 mM PBS; pH =7) with different initial starting potentials (E_{init}) Note: DPVs are offset for better visual interpretation cathodic peak dependence on potential..

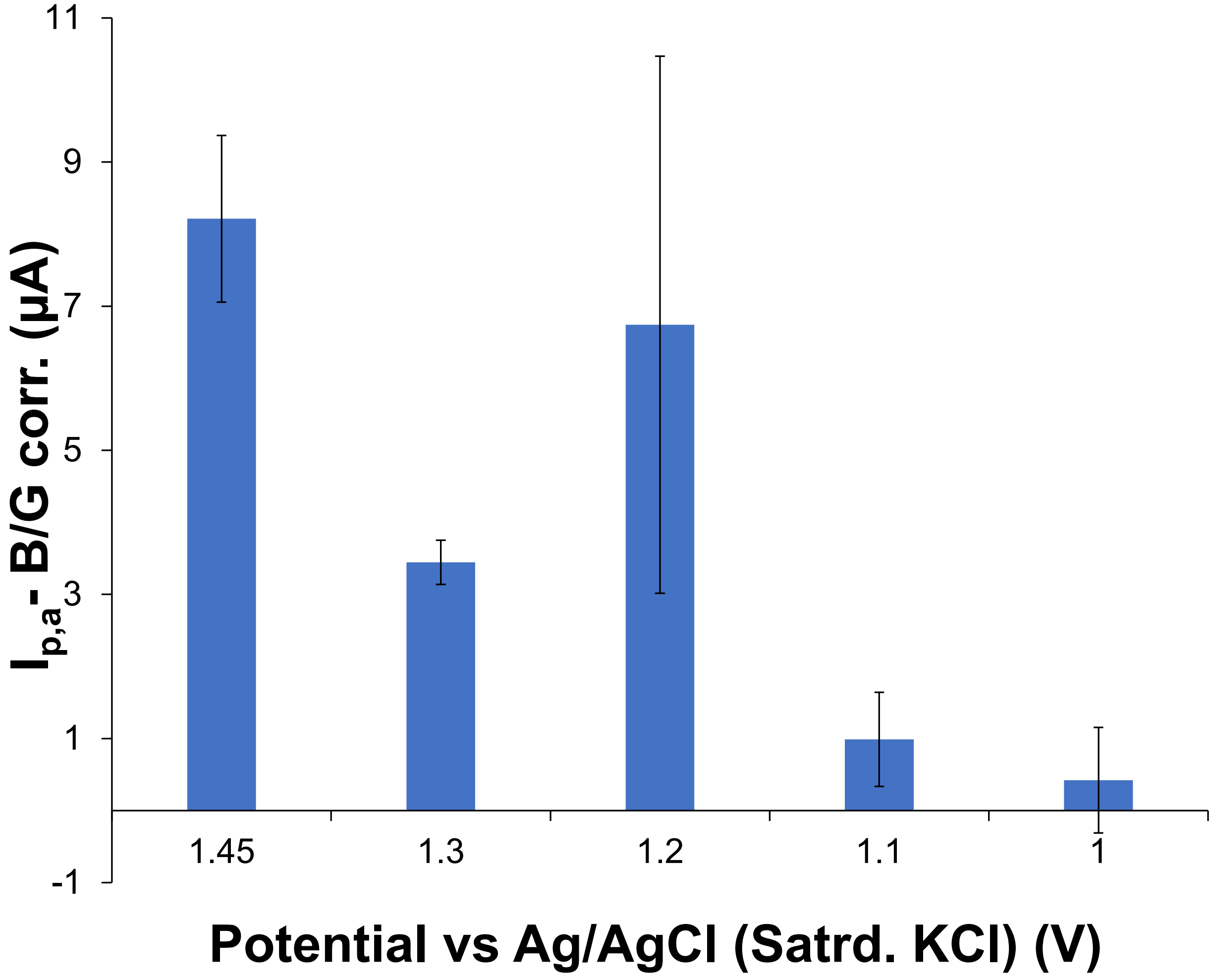

Figure S13. Tracking of $I_{p,c}$ as a function of E_{start} (n=3-5) for cathodic DPVs run with fully-modified electrodes with 75:25 HPU:TPU capping layers in 1 mM XYL. Note: Uncertainty is represented by standard deviation (n= 4).

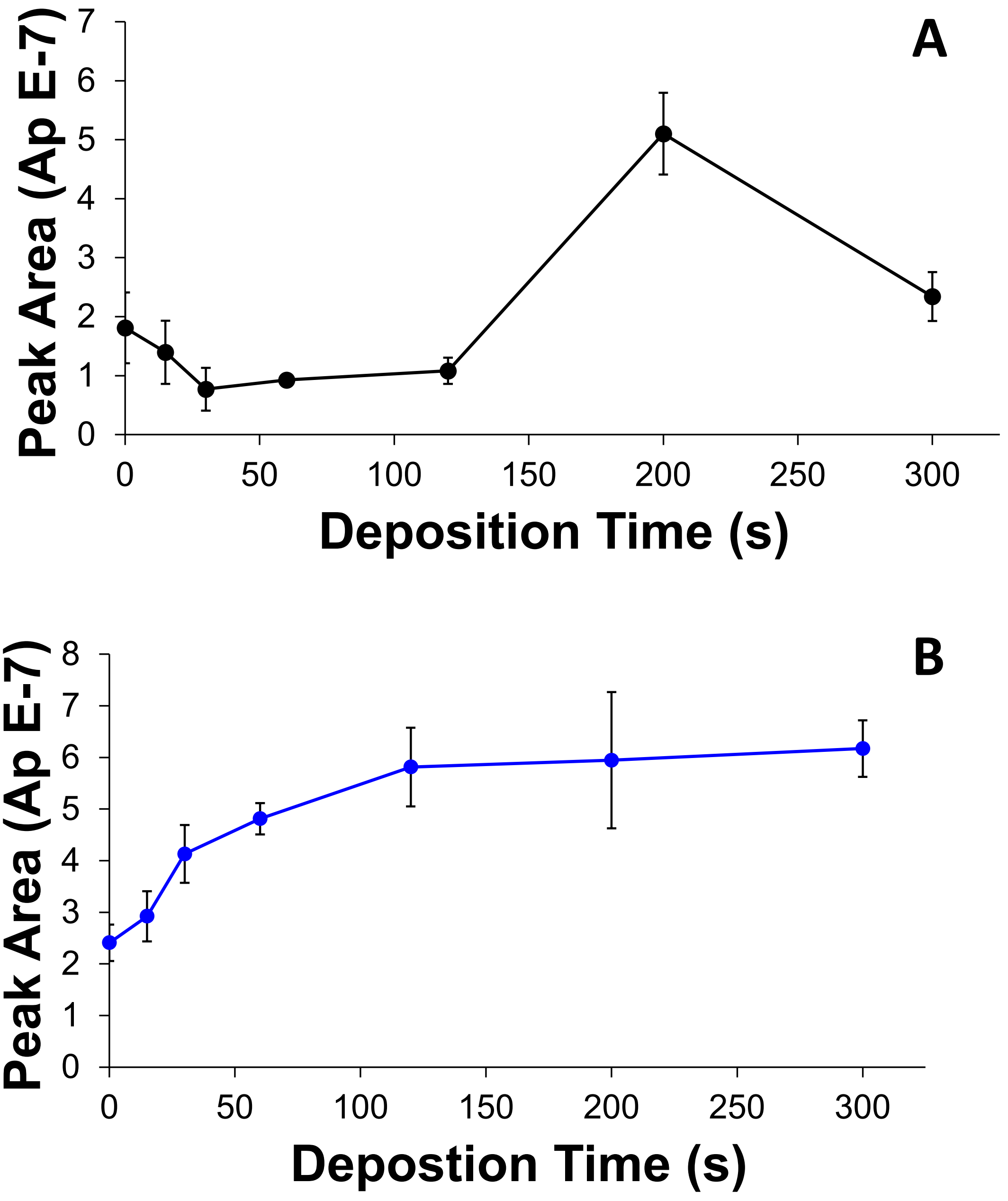

Figure S14. Tracking of the cathodic reduction peaks area during DPV stripping mode scans of (A) 75:25 and (B) 0:100 HPU:TPU capped modified electrodes in 300 µM XYL in PBS (150 mM; pH 7). Note: Uncertainty is represented with standard error (n=5).

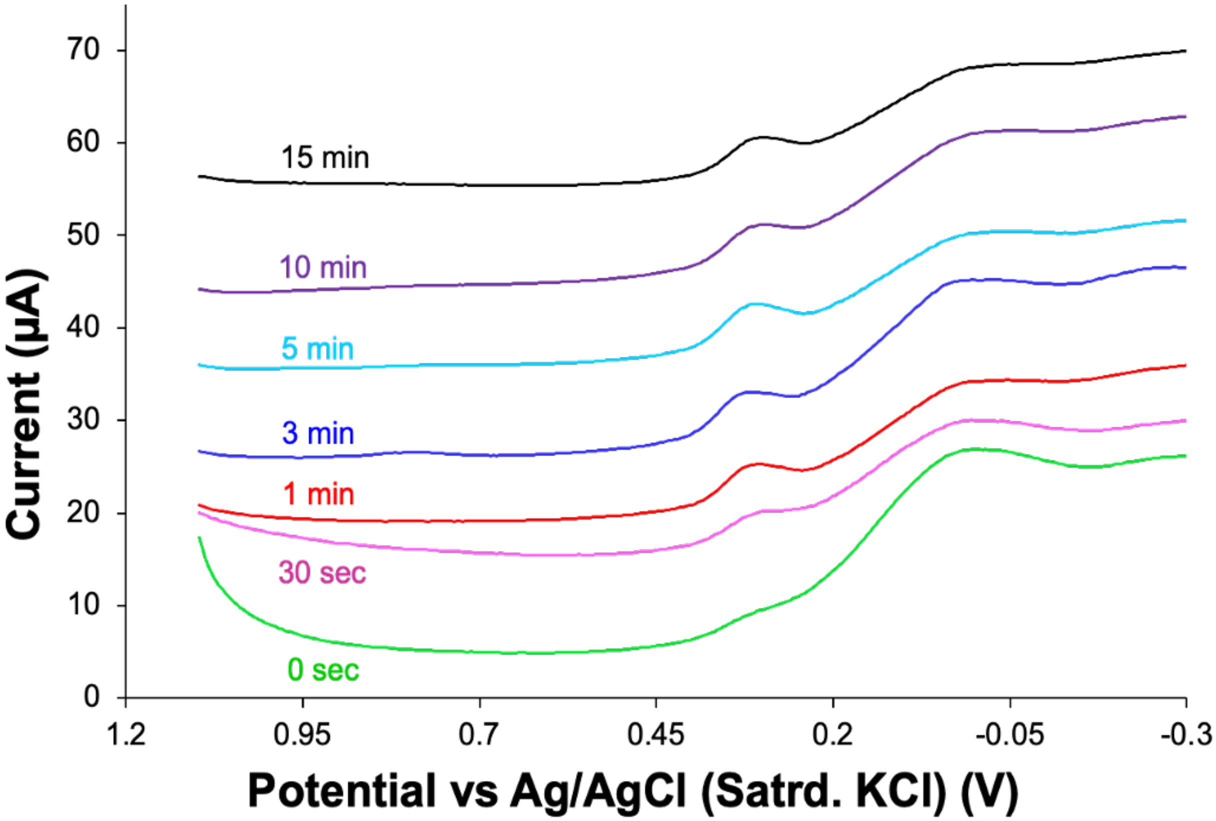

Figure S15. Representative cathodic DPSVs at of fully modified GCEs with 75:25 HPU:TPU capping layers immersed in 1 mM XYL (150 mM PBS; pH =7) with varying hold times at +1.1V prior to cathodic DPV sweep. Note: DPVs are offset on the y axis for better visual interpretation of cathodic peak dependence on time of initial applied potential before cathodic sweep.

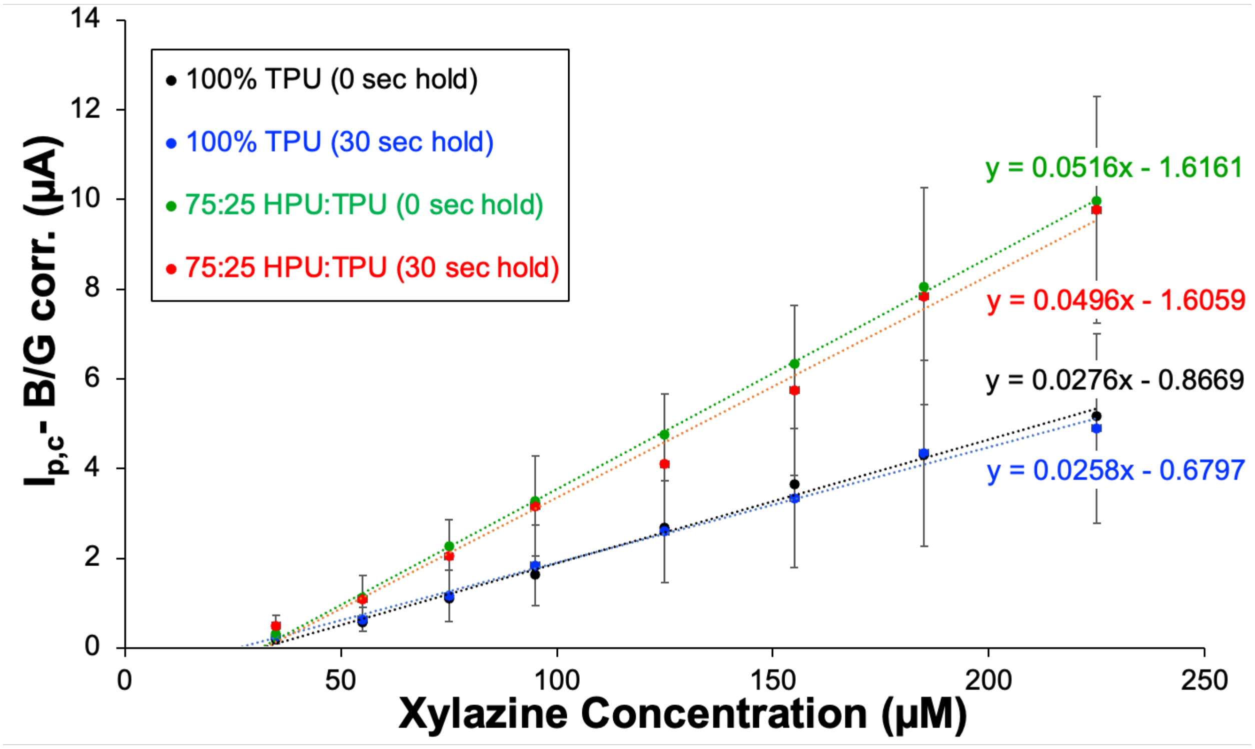

Figure S16. Calibration curve created from cathodic DPV responses (ip,c) with and without deposition hold times at +1.1V for both the 75:25 and 0:100 HPU:TPU capped modified electrodes. Uncertainty is represented by standard deviation (n=10,8,5,and 3).

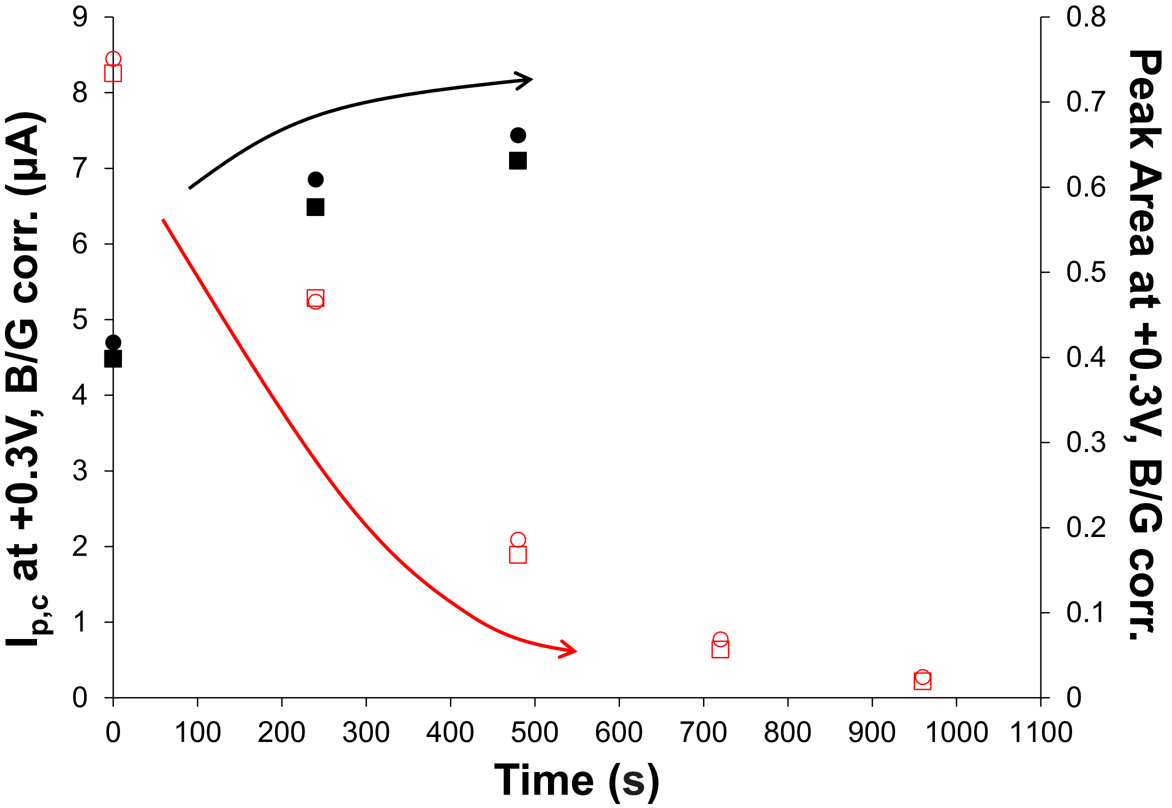

Figure S17. Tracking of both peak current ($I_{p,c}$) (*circles*) and peak area (*squares*) for 75:25 HPU:TPU capped electrodes kept in XYL (*solid symbols*) vs. electrodes transferred to PBS (*open symbols*) being immersed in 300 µM XYL solution for a 3-minute rest time after initial mixing (10 sec) and prior to applying a potential.

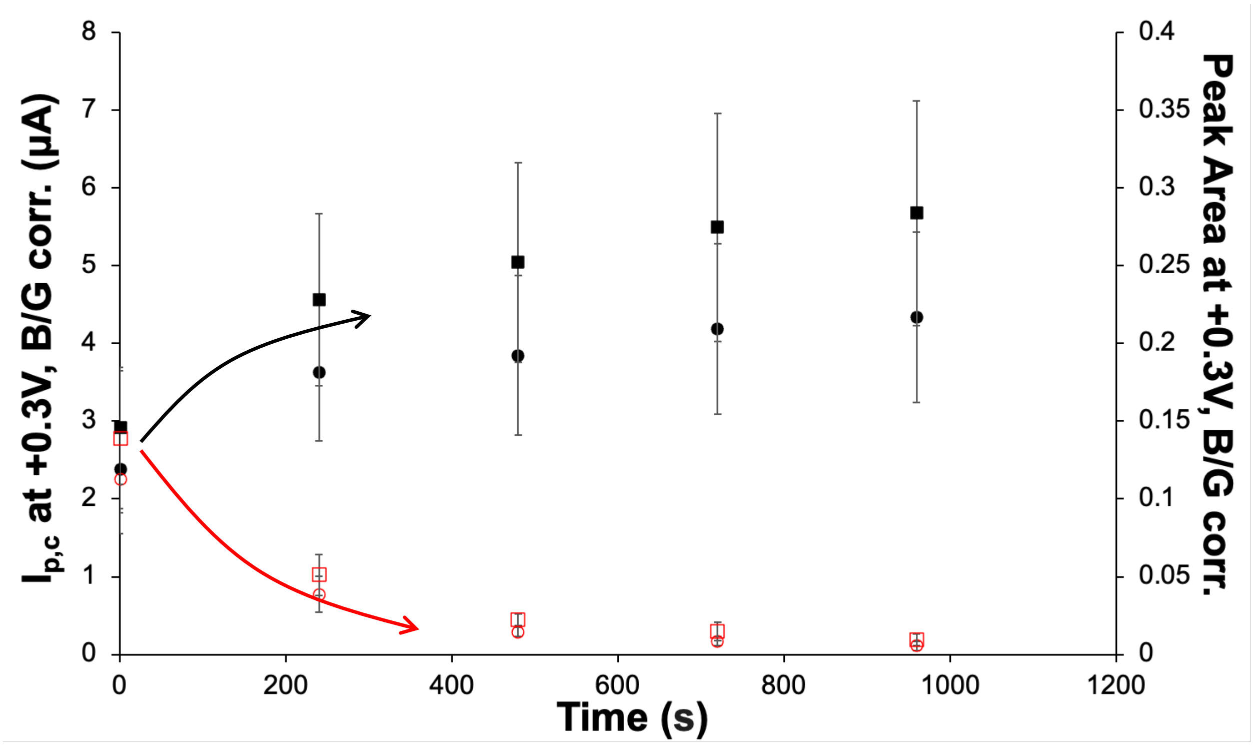

Figure S18. Tracking of both peak current ($I_{p,c}$) (*circles*) and peak area (*squares*) for 0:100 HPU:TPU capped electrodes kept in XYL (*solid symbols*) vs. electrodes transferred to PBS (*open symbols*) being immersed in 300 µM XYL solution for a 3-minute rest time after initial mixing (10 sec) and prior to applying a potential. Note: Uncertainty is represented with standard error (n=)

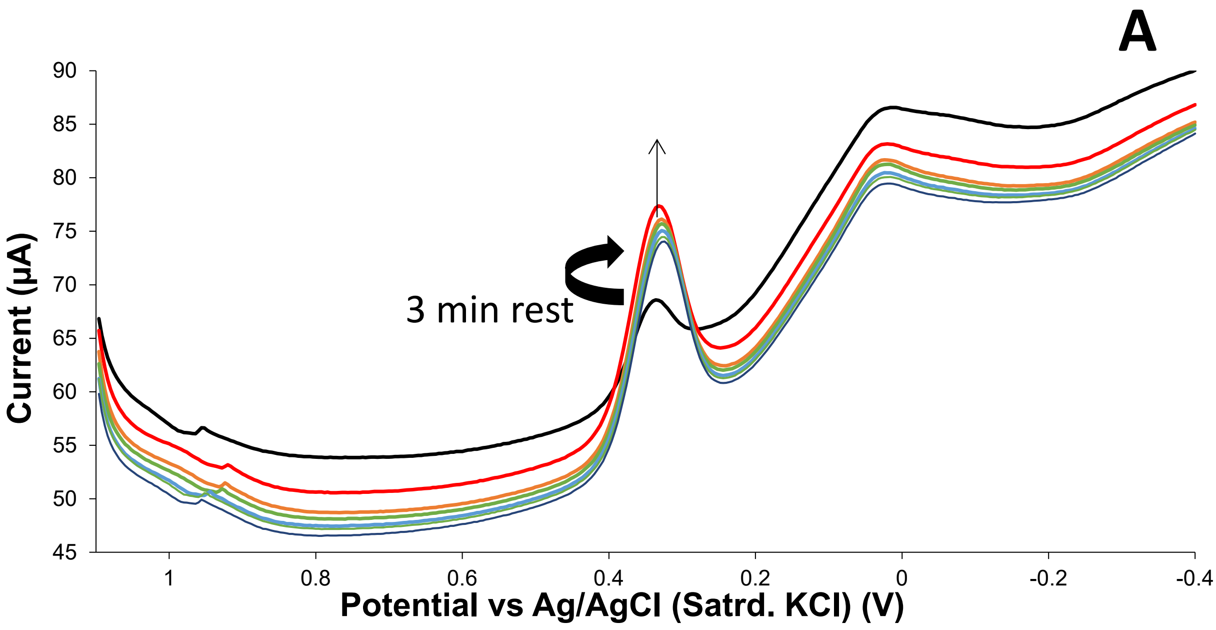

Figure S19. Fully-modified electrode (100% TPU capped) in 300 µM XYL (150 mM PBS at pH = 7) using 10 second stir followed by a 3-minute rest before establishing initial DPV signal (black traces) before **(A)** transferring the modified electrode to a 600 µM solution (Fig. 5A scheme (b)), stirring for 10 seconds followed by a 3-minute rest, and then continuously running sequential DPVs or; **(B)** transferring the modified electrode to a 600 µM solution and then continuously running sequential DPVs (no stir; no rest) for a closer inspection of the immediate XYL diffusion through the PU layer.

A

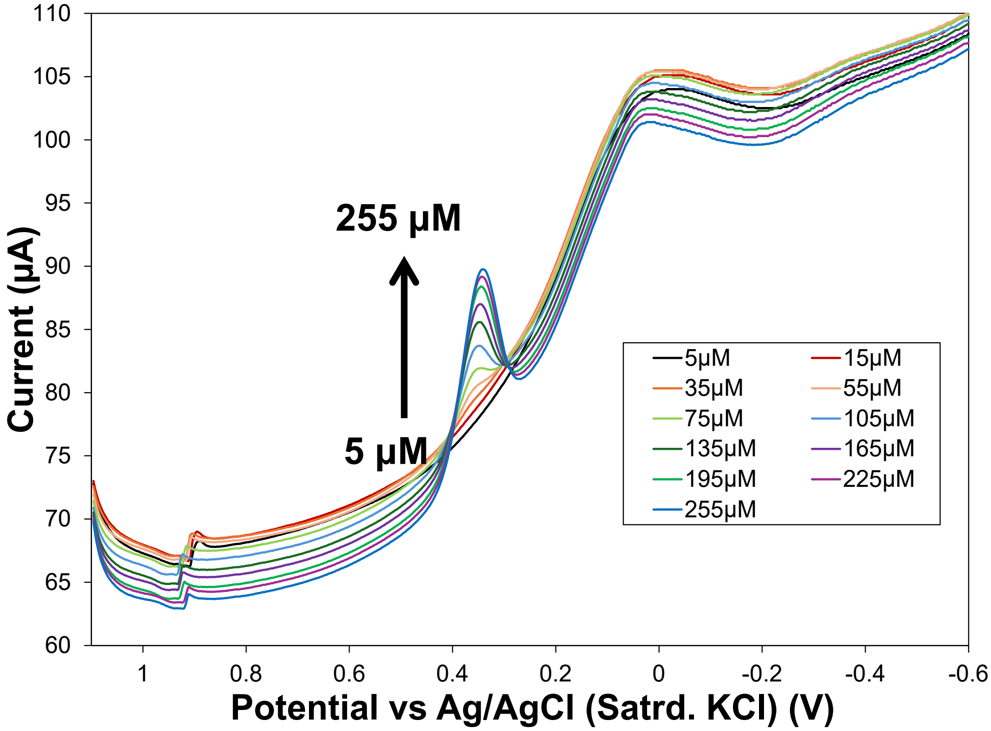

B

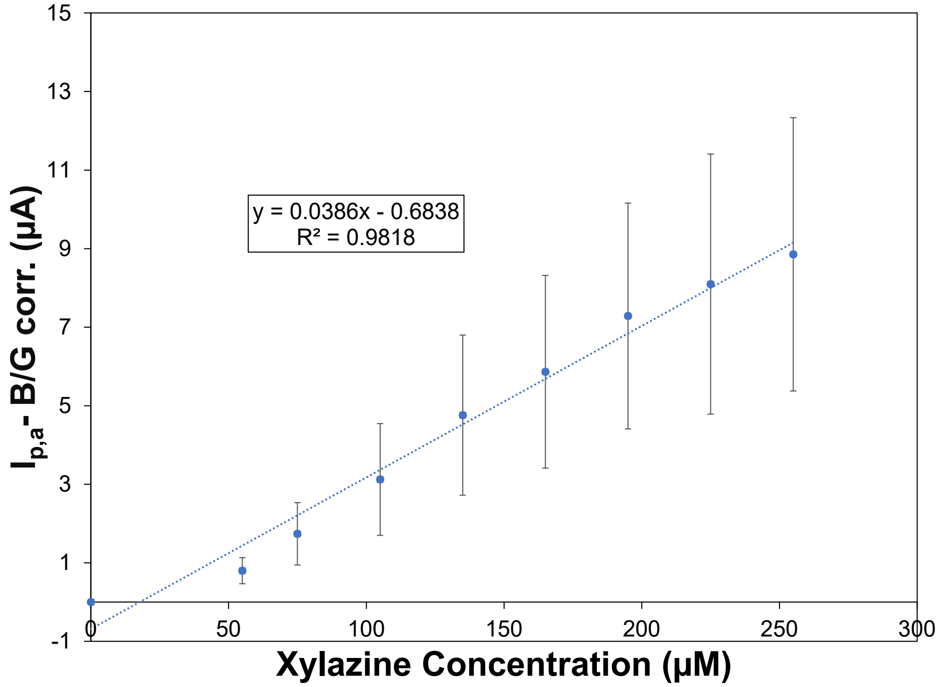

Figure S20. Cathodic DPV scans collected at the modified GCE utilizing the 0:100 HPU:TPU (100% TPU) capping layer in increasing concentrations of XYL standard (5 to 255 μM XYL in 150 mM PBS at pH = 7) and **(B)** corresponding calibration curve created from background corrected $I_{p,c}$ values (n=4) Note: Analogous results for the system capped with 100% TPU are provided in Supplementary Materials.

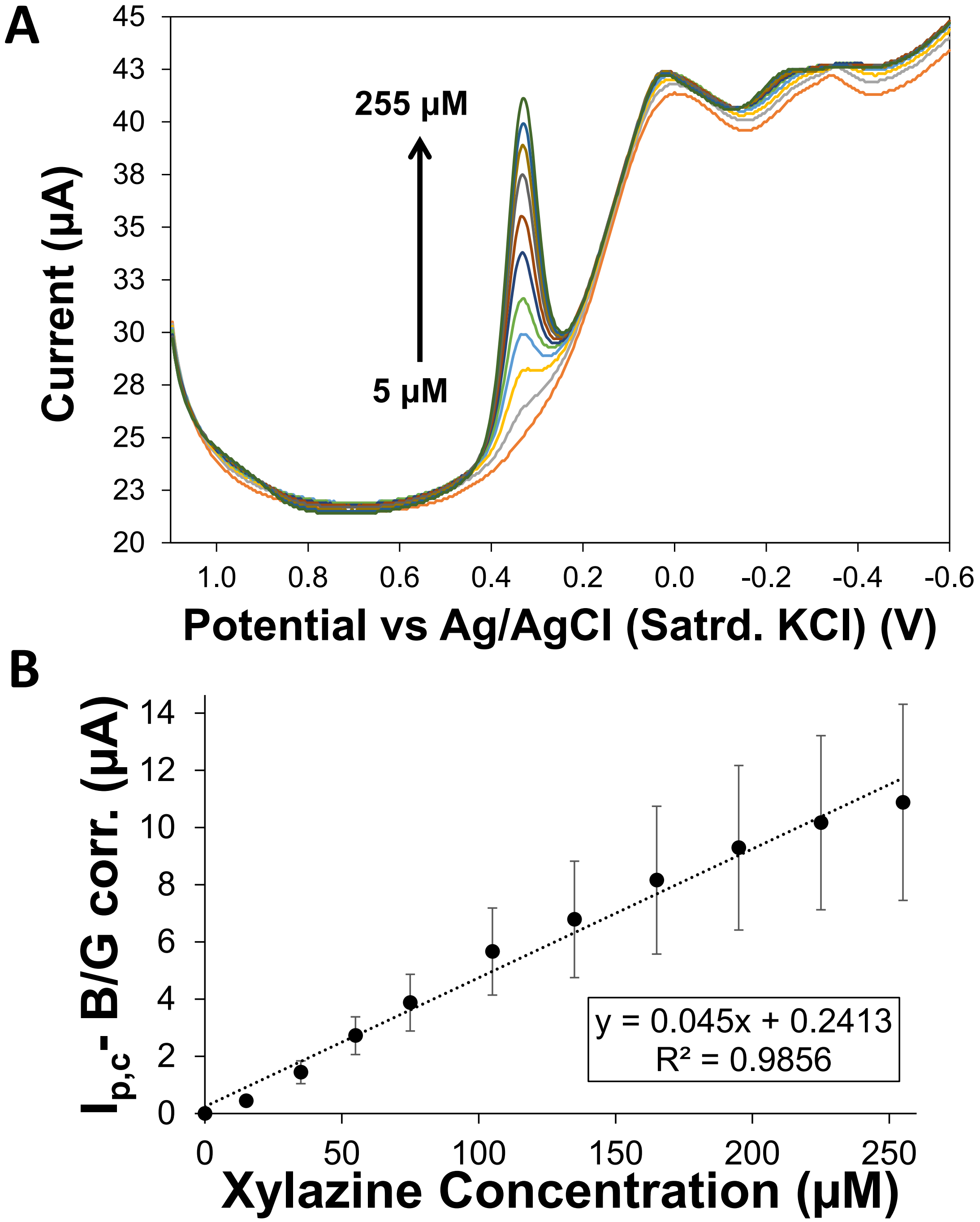

Figure S21. Cathodic DPV scans collected at the modified GCE utilizing the 75:25 HPU:TPU capping layer in increasing concentrations of XYL standard (5 to 255 μM XYL in 150 mM PBS at pH = 7) and **(B)** corresponding calibration curve created from background corrected $I_{p,c}$ values (n=3).

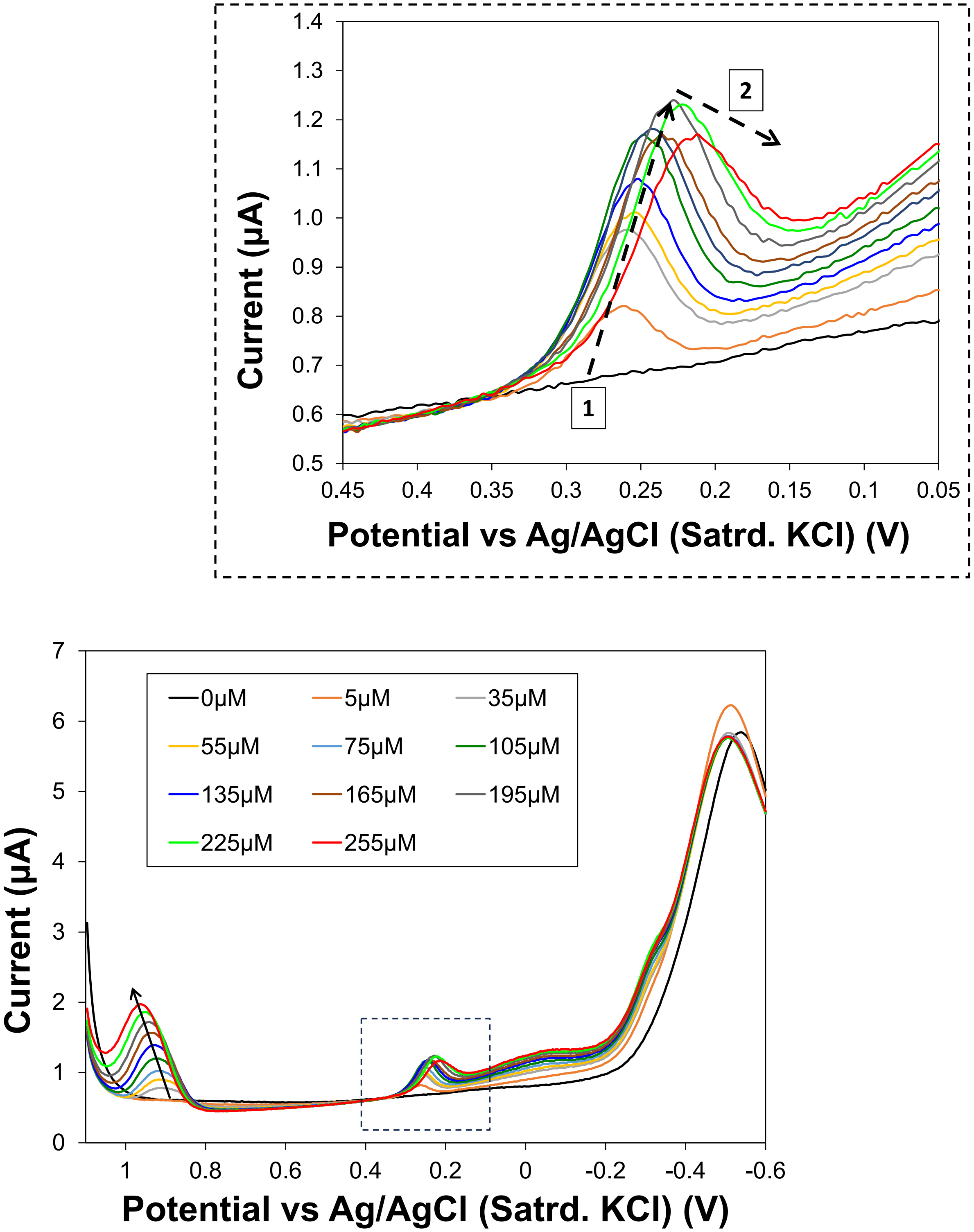

Figure S22. Cathodic DPV scans collected at the **un**modified or bare GCE in increasing concentrations of XYL standards (5 to 255 µM XYL in 150 mM PBS at pH = 7) with a E_{init} = +1.1V; corresponding expansion of the XYL oxidation product reduction voltametric peak (~+0.3 V) where $i_{p,c}$ **[1]** increases and shifts toward negative potentials at lower XYL concentrations and eventually **[2]** starts to decrease and shift more significantly at higher XYL concentrations. Note: 3 min stir followed by 3 min rest period.

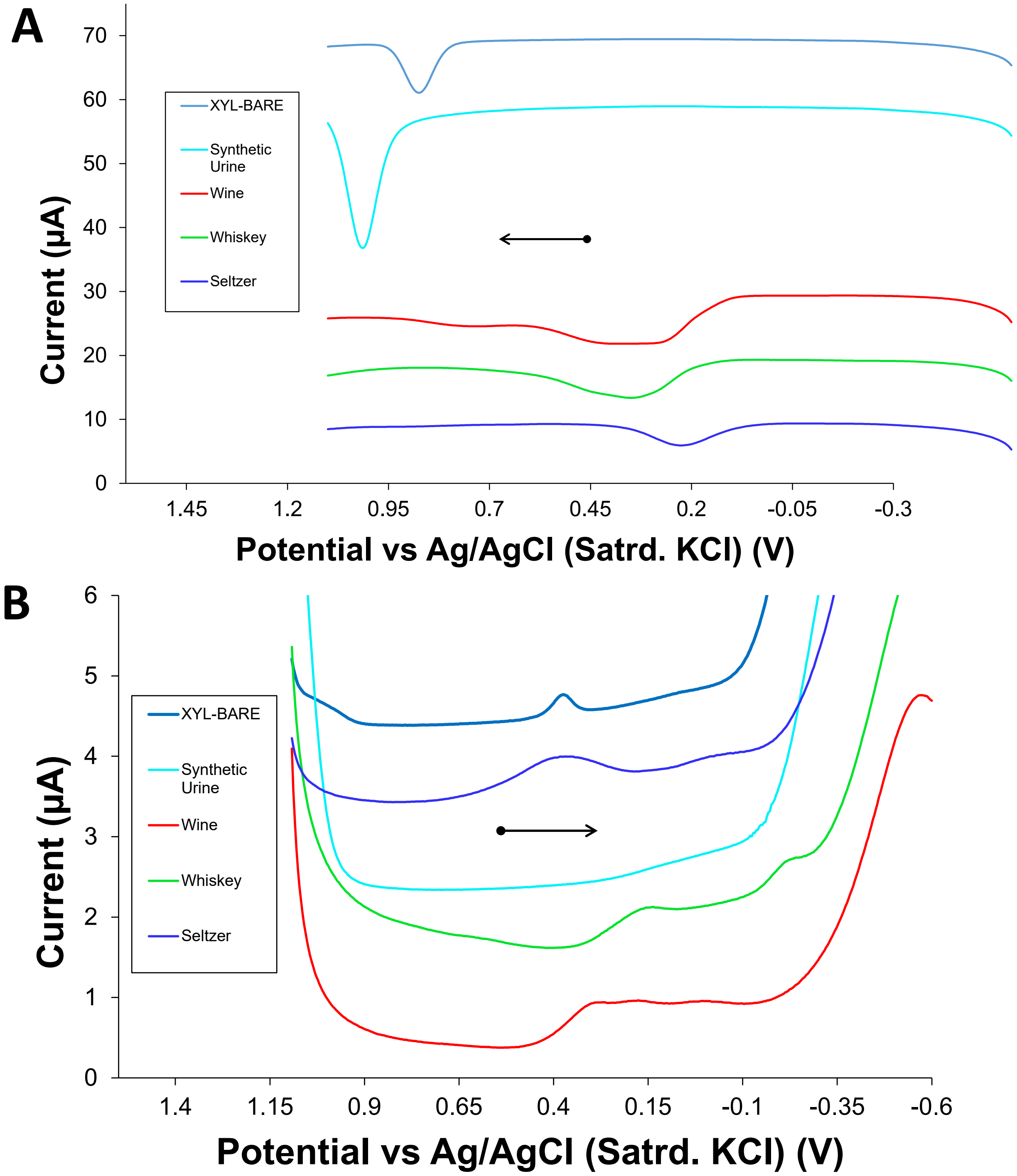

Figure S23. (A) oxidative/anodic and **(B)** reductive/cathodic DPV scans of bare (unmodified) GCE in solution of various matrices and 300 µM XYL. Note: Solutions were prepared by taking a 1:1 dilution with PBS.
